# Systematic Approach for Compound Angus Populations Revealing Positional Candidate Genes and Improving Prediction Accuracy in Carcass Traits

**DOI:** 10.3390/ani16172758

**Published:** 2026-09-02

**Authors:** Yuanyuan Yu, Yujiao Fu, Jiahong Zhao, Shiyu Wu, Shikai Wang, Zemin Li, Li Liu, Fang Sun, Jincheng Zhong, Jiabo Wang, Daoliang Lan, Yixi Kangzhu

**Affiliations:** 1 Key Laboratory of Qinghai—Tibetan Plateau Animal Genetic Resource Reservation and Utilization, Ministry of Education, Southwest Minzu University, Chengdu 610225, China; 2 Key Laboratory of Combining Farming and Animal Husbandry, Ministry of Agriculture, Institute of Animal-Husbandry, Heilongjiang Academy of Agricultural Sciences, Harbin 150028, China

**Keywords:** GWAS, GS, meta-analysis, carcass traits

## Abstract

Carcass traits, which reflect growth performance and muscle development, are economically important in beef cattle, yet their genetic determinants remain poorly characterized. Both single-population Genome-wide association studies (GWAS) methods, such as BLINK, and cross-population meta-analysis approaches are widely used to identify genetic variants, yet their comparative performance in genomic prediction for complex traits in structured populations remains underexplored. Few studies have directly compared these methods in genomic prediction. To address this gap, this study aims to (i) identify positional candidate genes associated with carcass traits and (ii) evaluate the context-dependent advantages of Covariate Adjustment (CA) and meta in genomic prediction. In this study, we analyzed carcass weight (CW), live weight (LW), and dressing percentage (DP) in 279 crossbred Angus cattle genotyped with the PHR0105_Bt140K_v1.0 SNP chip. GWAS was performed on the full population using BLINK, and results from three subpopulations were combined via meta-analysis, with significance thresholds for both approaches determined by a shuffle-based method. Candidate genes located within ±10 kb of significant SNPs were associated with different carcass traits, including *STRIT1*, *SEL1L3*, *NOC4L* and *ANK1* for DP; *SNCA* and *DNAH5* for CW; and *GYPC*, *GPR158*, and *GUCY1A1* for LW. Prediction accuracy under MAS and MABLUP showed meta slightly outperformed BLINK in MAS, while BLINK was better with covariate adjustment; after incorporating kinship in MABLUP, meta achieved higher accuracy and population partitioning was negligible. Overall, MABLUP yielded the highest accuracy (0.52–0.79) versus MAS (0.37–0.54) in all traits. These findings provide a methodological basis for selecting appropriate GWAS strategies in structured populations and highlight candidate genes.

## Simple Summary

Beef cattle production plays an important role in providing high-quality animal products, and improving economically valuable traits can help increase production efficiency and sustainability. However, the biological factors that influence important carcass characteristics are complex and are not fully understood. In this study, we investigated the genetic factors affecting carcass performance in a group of crossbred Angus cattle and compared different approaches for identifying useful genetic information and predicting animal performance. We identified several regions of the genome that were related to important carcass characteristics and found that combining information from different cattle groups could improve the reliability of genetic prediction in some cases. We also demonstrated that using information about genetic relationships among animals provided more accurate predictions of future performance. These findings improve our understanding of the genetic basis of beef production traits and provide useful information for selecting superior cattle, which may contribute to more efficient and sustainable beef production.

## 1. Introduction

With the continuous growth of the global population, beef consumption has increased steadily over recent decades [1]. Across the country, beef is the third most consumed meat after poultry and pork, highlighting its major role in overall meat supply [2]. Therefore, efficient beef production is crucial for maintaining a stable meat market and supporting the sustainability of the livestock sector. Given this context, carcass traits have become key indicators of beef production performance. Traits such as live weight, carcass weight, and dressing percentage directly determine beef yield and carcass quality. As classical quantitative traits controlled by multiple genes and shaped by environmental factors [3], identifying their genetic determinants is essential for improving breeding efficiency.

Angus is an important beef cattle breed widely used in commercial production and crossbreeding programs because of its favorable growth performance, carcass characteristics, and meat quality. Genetic studies in Angus and Angus-derived populations have identified variants associated with economically important production traits. Accordingly, considerable efforts have been made to characterize the genetic basis of economically important traits in Angus cattle. For example, Gill et al. (2010) identified several SNPs associated with carcass and meat-quality traits in a commercial Angus-cross population, indicating that genetic associations identified in Angus-derived populations may vary according to genetic background [4]. Subsequently, Seabury et al. (2017) identified genomic regions associated with growth and feed-efficiency traits, including loci affecting residual feed intake and growth-related phenotypes, and demonstrated that these loci could contribute to genomic selection for improved production efficiency [5]. More recently, Sanchez et al. (2023) integrated GWAS results from 54,782 animals representing 15 purebred and crossbred populations and showed that meta-analysis could identify additional QTL for growth and carcass traits compared with within-population GWAS, highlighting the value of combining genetic evidence across populations [6]. Kolpakov et al. (2025) conducted a GWAS in 260 Aberdeen Angus bulls and identified multiple SNPs associated with slaughter weight, meat yield, and meat marbling, including candidate variants located within or near *OSBPL3* and *RBFOX3* [7]. In a larger-scale study, Baneh et al. (2025) performed GWAS in 13,241 Angus cattle using more than 6.5 million imputed whole-genome sequence variants and identified multiple genomic regions associated with carcass weight, marbling score, rib-eye area, and backfat thickness [8]. Together, these findings demonstrate that economically important carcass traits in Angus cattle are influenced by numerous genomic loci, supporting a complex and polygenic genetic architecture. However, most previous studies have focused on individual or relatively defined populations, and less is known about the consistency of carcass-trait associations across Angus-derived populations with distinct genetic backgrounds, particularly populations containing both purebred Angus and Angus × Wagyu crossbred cattle. This population structure provides an opportunity to examine whether alternative GWAS strategies can effectively detect and integrate genetic signals across heterogeneous Angus-derived populations.

GWAS have been widely used to identify genomic variants associated with complex traits by exploiting linkage disequilibrium between genetic markers and causal variants [9,10]. Despite these advances, GWAS power can diminish in structured or composite populations, where differences in allele frequencies, genetic backgrounds, and linkage disequilibrium (LD) patterns may obscure true signals and increase false positives. Thus, single-population GWAS may be insufficient to capture the full genetic architecture of complex traits across diverse cattle populations.

Population stratification is a major challenge in GWAS, especially when individuals are collected from different data sources. In the traditional GWAS approach, subpopulation affiliation can be considered as a covariate effect. This method is called the approach within model or Covariate Adjustment (CA). For example, age, sex, farm, and principal components have been incorporated as covariates to control potential confounding effects in cattle GWAS [11,12]. In our study, subpopulation affiliation was encoded using binary indicator variables (1 = membership and 0 = non-membership) and included as a fixed-effect covariate in the joint GWAS model. The GWAS results generated from this CA model are uniformly designated as “index” throughout this study. Another approach is post-GWAS meta-analysis, which uses the GWAS results from every single subpopulation, then evaluates all markers’ contribution under heterogeneity from different sources [13]. There is no exact conclusion of which method is the best, especially in beef carcass traits.

The Bayesian information and Linkage disequilibrium Iteratively Nested Keyway (BLINK) method uses LD information to optimize covariates and Bayesian Information Criterion (BIC) to iteratively refine pseudo-quantitative trait nucleotides (QTNs) within a fixed-effect framework, effectively controlling population structure and reducing false positives [14,15]. While BLINK excels in analyzing individual GWAS datasets, integrating results across multiple populations requires a complementary approach. GWAS meta-analysis provides an alternative framework for integrating association evidence across multiple populations [16]. METAL is a widely used tool for efficiently combining GWAS summary statistics across cohorts [17].

To validate the effectiveness and utility of identified genetic markers, the most definitive approach is through gene knockout or targeted interference experiments. However, such methods are often impractical and ethically challenging in large animal studies. A more feasible alternative is to leverage marker-assisted selection (MAS) [18] or genomic selection (GS) [19,20] for predictive validation. In this context, the improvement in prediction accuracy contributed by significant markers in an independent validation population can serve as a key verification criterion. A truly associated and functional marker should explain a portion of the genetic variance and demonstrably enhance predictive performance in genomic selection models.

To overcome these limitations, we address two key research questions: (i) identifying candidate genes associated with beef carcass traits; and (ii) comparing and validating the predictive performance of SNPs detected by CA and meta-analysis in GS. The findings will not only evaluate their practical breeding utility but also provide methodological insights for optimizing multi-population GWAS strategies and enhancing genetic improvement in carcass traits.

## 2. Materials and Methods

### 2.1. Sample Collection and Genotyping

A total of 279 beef cattle was sampled from three locations in China: Sunwu County, Heilongjiang Province (48 Angus), Dachang Hui Autonomous County, Hebei Province (109 Angus × Wagyu crossbreds), and Qingyang City, Gansu Province (122 Angus). All animals were raised until 28–30 months of age and subsequently slaughtered. Phenotypic data for live weight (kg), carcass weight (kg), and dressing percentage (%) were collected following standardized carcass evaluation procedures, including electrical stimulation and inverted exsanguination. Live weight was measured after 6 h fasting period. Carcass weight was recorded after removing the internal organs, and dressing percentage was calculated as carcass weight divided by live weight. Blood and muscle samples were collected, and information on age, ear tag number, and geographic origin was recorded for subsequent genotyping analyses. All individuals’ information were recorded in both of notes and Excel files as independent and verifiable backups.

Genomic DNA was extracted from muscle and blood samples using the GenoPrep^®^ DNA Extraction Kit (magnetic-bead method; Boready Biomarker Technologies, Shijiazhuang, China). DNA concentration and integrity were assessed using a Qubit fluorometer and 1% agarose gel electrophoresis, respectively. Genomic libraries were prepared and genotyped by Boready Biomarker Technologies using the GenoBaits^®^ Bovine 140K Panel (product ID: PHR0105_Bt140K_v1.0). Briefly, 500 ng of genomic DNA was enzymatically fragmented, followed by end repair and A-tailing at 37 °C for 20 min and 72 °C for 20 min. Sequencing adapters containing sample-specific barcodes were then ligated in a 40 μL reaction at 22 °C for 60 min, followed by magnetic-bead purification and high-fidelity PCR amplification. For target enrichment, 500 ng of the prepared library was hybridized with the GenoBaits^®^ Panel, Block I, and Block II at 65 °C for 2 h. The hybridized fragments were captured using GenoBaits^®^ DNA Probe Beads, washed to remove non-target fragments, and subsequently PCR-amplified and purified. The enriched libraries were sequenced, and genotypes at the targeted SNP loci were called and exported in HapMap format. Quality control was conducted in R(v4.3.3), and SNPs were removed based on the following criteria: (1) missing rate > 0.05; (2) minor allele frequency (MAF) < 0.01; (3) presence of indels; and (4) multiple alleles. After filtering, genotype imputation was performed using Beagle (v4.1) [21], and high-quality SNPs were retained for subsequent GWAS and GS analyses.

### 2.2. Population Stratification

The Genome-Associated Prediction Integrated Tool (GAPIT) [22] implemented in R (v4.3.3) was employed to conduct a comprehensive set of genomic analyses, including linkage disequilibrium (LD) and heterozygosity assessments among markers, as well as population structure analyses through principal component analysis (PCA) and phylogenetic relationship reconstruction using a neighbor-joining (NJ) tree. Moreover, genome-wide LD decay curves were subsequently generated using PopLDdecay (v3.42) to calculate LD and summarize its decay with increasing inter-marker distance [23].

### 2.3. Association Study

To establish an appropriate significance threshold, the permutation test was applied to determine the genome-wide threshold line. Phenotypic values for the CW were randomly shuffled among individuals to break the true genotype–phenotype associations, and GWAS was repeated 50 times. The minimum *p*-value from each permutation was recorded. The 5th percentile of the resulting distribution of minimum *p*-values was used as the empirical genome-wide significance threshold. Given that a sufficient number of permutation shuffles effectively stabilized the false-positive bias arising from the random distribution of genotypes and phenotypes, and that the distributions across multiple traits were highly similar, we chose the CW trait for permutation-based threshold determination of significant GWAS loci in this study. The same threshold was therefore applied consistently to LW, DP, and the meta-analysis to maintain a uniform significance criterion.

In this study, genome-wide association analysis was performed using the BLINK model implemented in the GAPIT (v4.0). BLINK is a multi-locus method that iteratively incorporates associated markers as covariates while testing additional markers, thereby enhancing statistical power and reducing false positives [14]. The BLINK model was run with Random.model = FALSE and Multi_iter = FALSE. The BLINK model can be expressed as follows:

(1) $$Y=PCA+{SNP}_{i}+e$$

(2) $$Y=PCA+QTNs+{SNP}_{i}+e$$

where Y is the phenotype vector, and *PCA* denotes the top principal components fitted as fixed effects to account for population structure. *QTNs* represents the set of associated markers selected in previous iterations and included as covariates. *SNP_i_* is the genotype of the marker being tested, and *e* is the residual error, assumed to follow a normal distribution.

BLINK employs two fixed-effect models: one for selecting QTNs and another for testing marker effects, which enhances both statistical power and computational efficiency. Model convergence is determined using the BIC, ensuring more accurate model selection compared with likelihood-based approaches. Manhattan and quantile–quantile plots were generated using GAPIT’s built-in functions to visualize the distribution and significance of association signals.

### 2.4. Meta-Analysis

For the meta-analysis, we employed the z-score (sample size-based) method implemented in the METAL (version 2020-05-05) software. This approach combines different parameters from individual studies, including sample size, *p*-values (p*i*), and the direction of effects. For each variant, the method converts the *p*-value into a z-score using the formula.

(3) $$Z=\frac{\sum_{i}Z_{i}w_{i}}{\sqrt{\sum_{i}w_{i}^{2}}}$$

where $W_{i}$ = $\sqrt{N_{i}}$ represents the weight (square root of the sample size) for study *i*, and$\;Z_{i}$ is the study-specific Z-score, calculated as

(4) $${\;Z}_{i}=\Phi^{-1}\left(1-p_{i}/2\right)\times sign\left({∆}_{i}\right)\;$$

Here, $\Phi^{-1}$ denotes the inverse cumulative distribution function of the standard normal distribution and *p_i_* is the *p*-value of the *i*th study. ${∆}_{i}$ is the estimated effect of the variant in the *i*th study, and sign(Δi) represents the direction of the effect.

The overall *p*-value was calculated as

P = 2 Φ (−|Z|)(5)

where Φ is the cumulative distribution function of the standard normal distribution.

This method ensures that larger studies contribute more heavily to the combined test statistic, thereby weighting the different studies according to their sample size. The significance threshold for the meta-analysis was defined consistently with that used in the GWAS.

### 2.5. Gene Annotation

Genes within ±50 kb regions surrounding significant SNPs were annotated based on the bovine reference genome Bos_taurus.ARS-UCD2.0.114.gff3 (https://ftp.ensembl.org/pub/release-114/gff3/bos_taurus/ (accessed on 24 December 2025)). Genes located within the annotated regions of significant SNPs were considered positional candidate genes, while the LD patterns surrounding the significant SNPs were used to further characterize and prioritize candidate loci. We converted genotype files in HapMap format to VCF format using TASSEL (v5.0) [24], which was then used for subsequent LD analysis. For each significant SNP, all SNPs within ±500 kb upstream and downstream were extracted, and the corresponding genotype data were formatted as VCF files for input into LDBlockShow (v1.41) [25]. LDBlockShow calculated pairwise LD metrics, such as r^2^ or D’, between SNPs and automatically defined LD blocks, providing a visualization of the linkage relationships and block structures within the candidate gene regions.

### 2.6. Genomic Selection

In this study, we conducted GS predictions using two approaches. First, MAS was implemented using GWAS-derived significant SNPs as predictors. The significant SNPs were incorporated into linear models together with population-related covariates (index and the first three principal components (PCs)), allowing us to evaluate the predictive power of top associated loci for the traits of interest. Second, a mixed-model approach incorporating both the kinship matrix and the significant SNPs—referred to as marker-assisted best linear unbiased prediction (MABLUP)—was applied. MABLUP used the same fixed effects as MAS, while additionally incorporating the genomic kinship matrix to model the random additive genetic effect. This approach integrates genome-wide genetic relationships with trait-associated markers to improve genomic prediction. By using these approaches, we aimed to evaluate the effectiveness of SNPs discovered through CA and meta-analysis for genomic selection prediction.

For the MAS model, populations from Sunwu, Qingyang, and Dachang County were each subjected to five-fold cross-validation. In each fold, the selected training sets were combined, and the remaining individuals formed the test set. GWAS was conducted on the training set, with the index and PCs included as a covariate to identify significant SNPs. In the test set, the significant SNPs identified from the training set were included as fixed effects together with PCs in a linear model (LM) to predict phenotypes and genomic estimated breeding values (gEBVs). Prediction accuracy was assessed by calculating the Pearson correlation between the predicted values and the observed phenotypes. If no significant SNPs were identified in the training set, only PCs was used as fixed effects. Thus, both SNP selection and model fitting were restricted to the training set within each cross-validation fold, whereas the test set was used only for independent prediction and evaluation, thereby avoiding information leakage. In the meta part, GWAS was conducted separately for the three training sets without including the index as a covariate. The results were then combined in METAL to identify significant SNPs. Subsequent prediction in the test set followed the same procedure as in CA, using the significant SNPs together with PCs as fixed effects in the LM to estimate phenotypes and gEBVs, and calculate prediction accuracy.

The prediction linear model can be expressed as

(6) Y=μ+Xb+QTNα+e

where Y is the vector of observed phenotypes, *μ* is the overall mean, *Xb* represents additional fixed effects, including PCs. *QTN* contains the genotypes of the significant SNPs identified by GWAS, α is the vector of SNP effects, and *e* is the residual error. For the MAS and meta-analysis approaches, gEBVs were calculated as the predicted genetic values from the fitted model, including the contributions of PCs and significant SNPs.

For the MABLUP model, the division of training and test sets and the procedure for identifying significant SNPs were the same as in MAS. The significant SNPs identified by GWAS were included as fixed effects, while the kinship matrix was included as a random effect in MLM framework. MABLUP was implemented using the gBLUP procedure in GAPIT, with a VanRaden genomic kinship matrix and PCs as covariates. For MABLUP, gEBVs were obtained from the predicted genetic values including the effects of significant SNPs, PCs, and the kinship-based polygenic effect. The procedure for selecting significant SNPs was the same as in MAS. The model is formulated as

(7) Y=μ+Xb+QTNα+Zg+e

The parameter definitions are the same as in MAS, where (*Zg*) represents the random effects associated with the kinship matrix. Prediction accuracy was evaluated by the Pearson correlation between the gEBVs and the observed phenotypes.

In addition, we performed predictions using randomly divided populations into three equal subsets, following the same procedure for both MAS and MABLUP models. This experiment aimed to test whether the population structure adjustment can effectively improve prediction accuracy and to provide a finer comparison of CA and Meta prediction performance.

## 3. Results

### 3.1. Descriptive Statistics and Genetic Parameters Estimations

**Figure 1:**
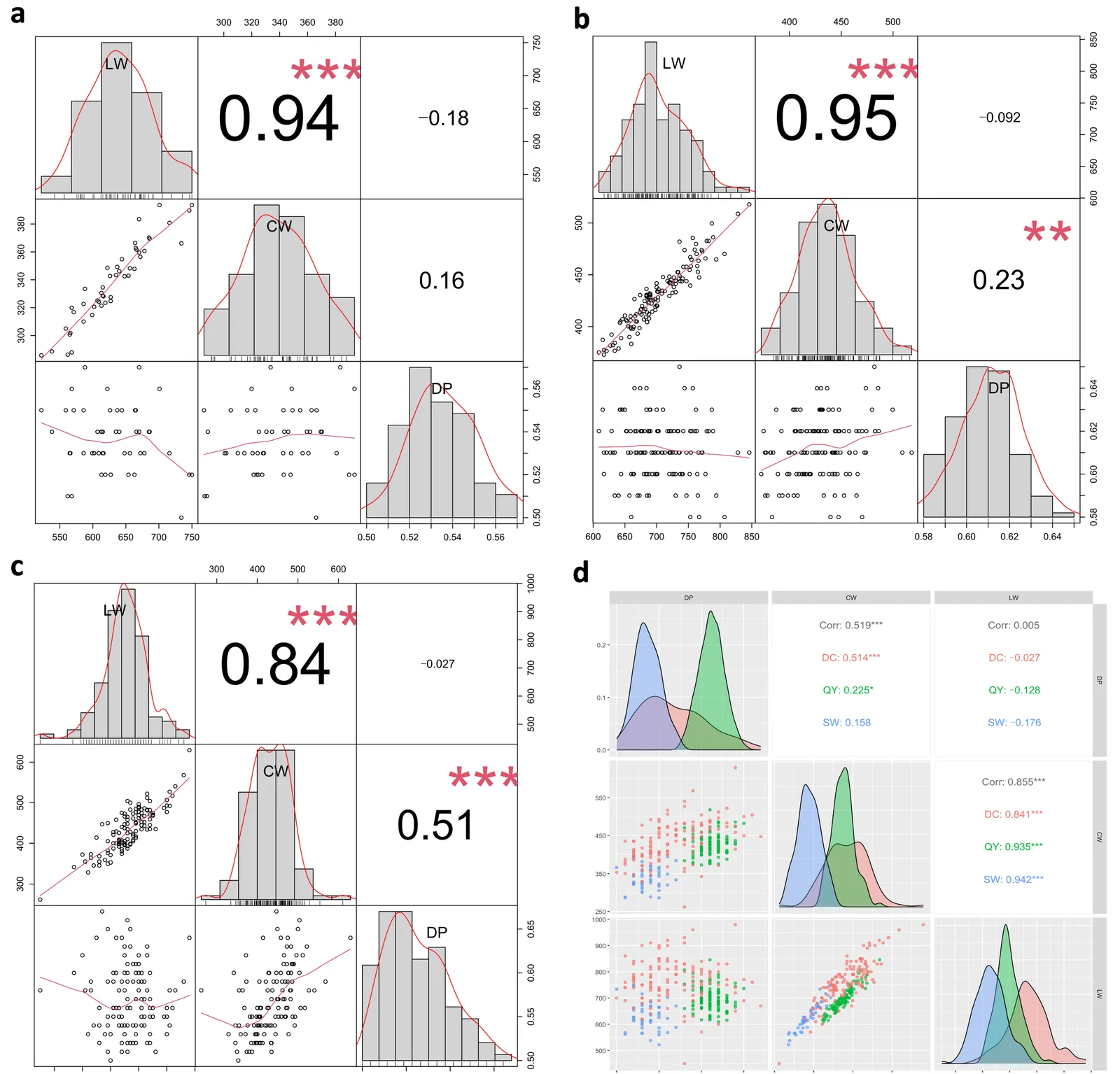
Correlation and distribution analysis of three carcass traits: live weight (LW), carcass weight (CW), and dressing percentage (DP). Pairwise phenotypic correlations were assessed using Pearson’s correlation coefficients within three distinct cattle populations: Sunwu (SW) (**a**), Qingyang (QY) (**b**), and Dachang (DC) (**c**). Each panel displays the kernel density plots along the diagonal, scatterplots in the lower triangle, and correlation coefficients in the upper triangle. Significance levels are denoted as * *p* < 0.05, ** *p* < 0.01, and *** *p* < 0.001 (**a**–**d**). Comparative distribution of LW, CW, and DP across the three populations (SW in blue, QY in green, and DC in red). Diagonal panels show trait-wise kernel density estimates, while the lower triangle displays pairwise scatterplots colored by population. The upper triangle summarizes Pearson’s correlation coefficients between traits within each population (**d**).

We analyzed three carcass traits in 279 cattle across three populations and summarized the descriptive statistics (Table S1). Most records of LW ranged from 450 kg to 980 kg. The mean value of LW, CW, and DP was 715.3 kg, 414.5 kg, and 57.9%. Specifically, the mean values for LW, CW, and DP were 766.39 kg, 434.70 kg, and 56.74% in DC; 695.41 kg, 425.33 kg, and 61.18% in QY; and 630.60 kg, 338.39 kg, and 53.62% in SW, respectively. All traits exhibited normal distributions. Pearson’s correlation coefficients were calculated for the three populations SW, QY and DC (Figure 1a–c). Across these populations, a strong positive correlation was detected between LW and CW, a moderate positive correlation between CW and DP, and a negative correlation between LW and DP. To further examine inter-trait relationships and group-specific patterns, pairwise correlation analyses and density distributions were explored (Figure 1d), yielding results consistent with those observed in the three individual populations. Group-specific density plots reveal distinct phenotypic distributions, particularly for DP, indicating possible population structure or differential selection. These population-specific phenotypic distinctions reflect potential differences in breeding objectives and environmental adaptation—for instance, QY exhibits superior dressing efficiency despite having a moderate body weight. Such pronounced phenotypic stratification further underscores the necessity of controlling for population structure in the subsequent GWAS to prevent spurious associations.

Genotyping of 279 cattle was conducted using Genotyping by Target Sequencing (GBTS) [26], targeting approximately 140K SNPs across the bovine genome. After quality control filtering to exclude SNPs containing indels, missing data, or multiple alleles, a total of 127,918 high-quality SNPs were retained for downstream analyses. For individual heterozygosity, the values were primarily clustered between 0.3 and 0.4, showing a distinct peak in this interval. In contrast, the heterozygosity of SNP markers was predominantly concentrated within 0.4–0.6, with the highest frequency observed in this range. The MAF exhibited a distribution spanning 0.01 to 0.5, with frequencies gradually increasing across this range (Figure S1).

We analyzed the correlation coefficient (R) and determination coefficient (R^2^) between the neighbor SNPs pairs in the genotype matrix. The upper panels show the distribution of raw correlation coefficients (R), which remained a stable decline with increasing distance (Figure S2). The lower panels display LD decay among markers, with R^2^ decreasing as distance increased and reaching a plateau at approximately 8 kb with a value around 0.3. The overall LD decay patterns were highly similar among DC, SW, and QY, with only minor differences observed beyond 250 kb, suggesting comparable recombination rates and genetic diversity (Figure S3).

### 3.2. Population Structure and Estimated Heritability

**Figure 2:**
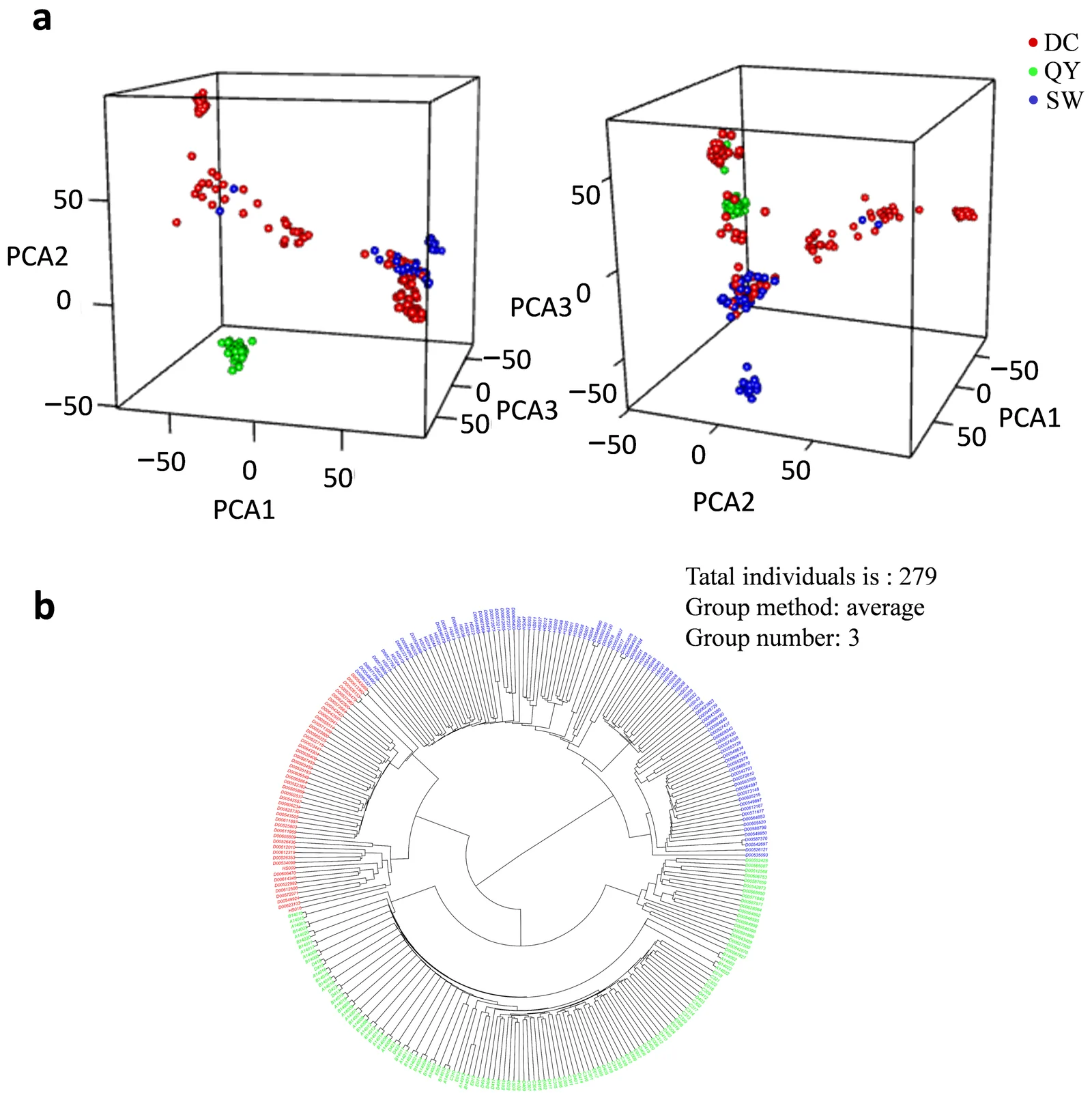
Genetic structure and phylogenetic relationships among individuals from Dachang (DC), Qingyang (QY), and Sunwu (SW). (**a**) PCA of genome-wide SNP genotypes, shown from two viewing angles derived from a three-dimensional PCA space. Each point represents an individual, with DC in red, QY in green, and SW in blue. The PCA reveals a distinct clustering of QY individuals, while DC and SW show partially overlapping distributions, indicating a higher degree of genetic affinity. (**b**) Neighbour-joining (NJ) tree constructed using pairwise genetic distances. Tip colors represent clustering results assigned by GAPIT based solely on genetic relatedness, and therefore do not correspond directly to the color scheme used in panel (**a**). The tree topology recapitulates the PCA pattern, showing QY as an independent lineage, while DC and SW cluster on adjacent branches, suggesting that they may share parts of an ancestral genetic background.

Visualization of the first three principal components revealed three distinct clusters, with individuals within each cluster showing close genetic relatedness. Interestingly, cattle from DC and SW were genetically more similar, reflecting geographic proximity. The first three principal components accounted for 9.96%, 5.7%, and 2.24% of the total genetic variance, respectively, highlighting the moderate but discernible population differentiation (Figure 2a). Although this proportion was relatively modest, the first three PCs clearly separated the three major populations, while the remaining variance was distributed across additional PCs, likely reflecting within-population diversity and finer-scale structure. The first five and ten PCs explained 20.78% and 24.82% of the cumulative variance, respectively (Figure S4), consistent with previous reports in Angus and Chinese cattle, where the first three PCs explained 14.43% and PC1 explained 9.56% of the genetic variation, respectively [27,28].From the perspective of the NJ tree, the genetic structure of the populations revealed that QY formed an independent lineage, while DC and SW clustered on adjacent branches, consistent with partial shared ancestry (Figure 2b).

The observed population structure revealed genetic differences among the three populations, with genetic variation also present within populations. Accordingly, variance components and heritability were estimated for the three carcass traits. Additive genetic variance accounted for a substantial proportion of the phenotypic variance for all three carcass traits (Table S2). The estimated narrow-sense heritability was 0.68 for LW, 0.67 for DP, and 0.61 for CW, indicating relatively similar levels of additive genetic control across traits. Correspondingly, the residual variance accounted for 31.6%, 33.4%, and 39.3% of the phenotypic variance for LW, DP, and CW, respectively.

### 3.3. GWAS, Meta-Analysis, and Candidate Gene

**Figure 3:**
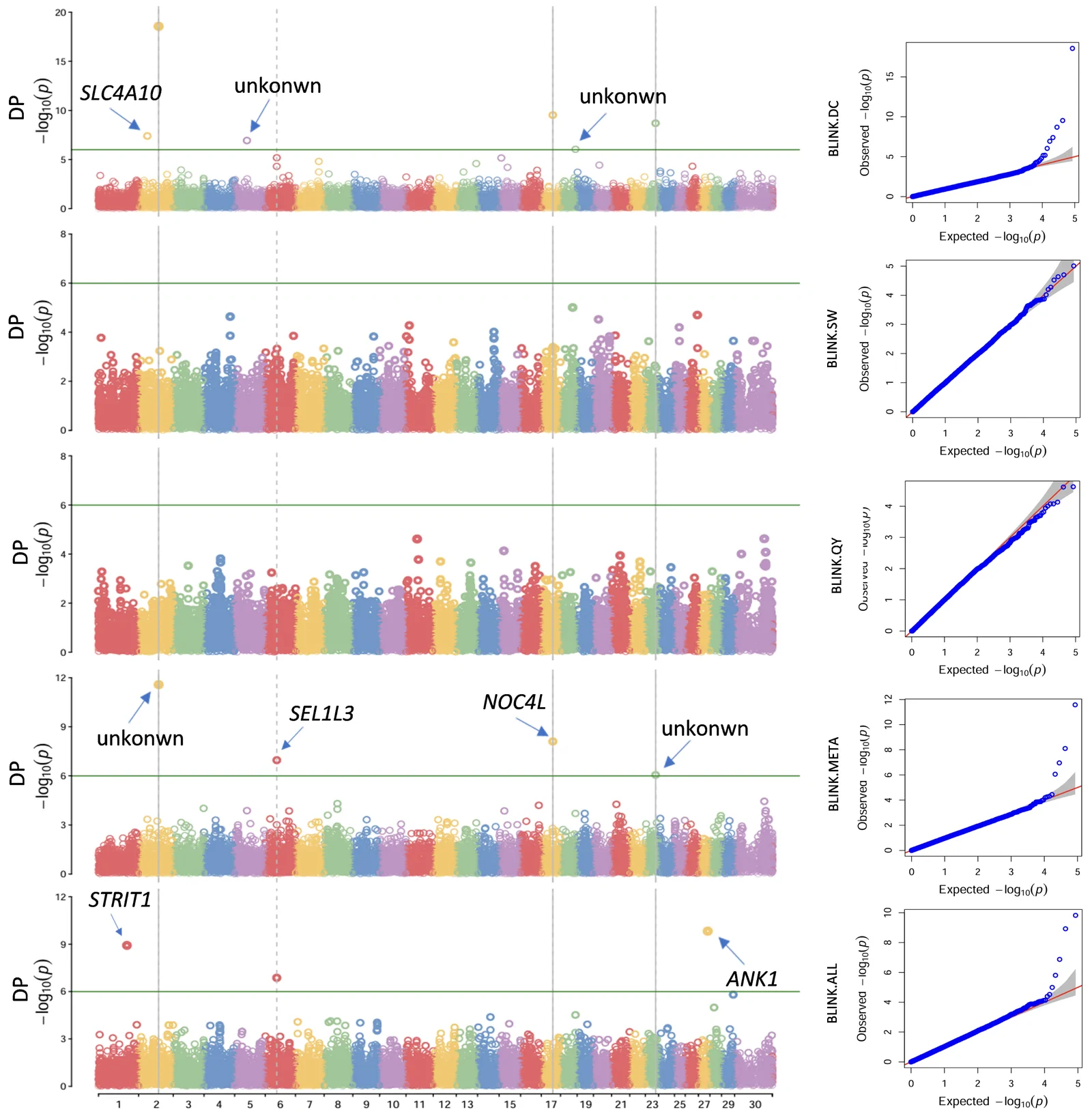
Manhattan and quantile–quantile (QQ) plots of the *p*-values for the genome-wide association study of the DP trait in cattle, performed separately for the Sunwu, Dachang, and Qingyang populations, for the combined population, and for the meta-analysis, based on the BLINK method; the horizontal line of significance threshold (*p*-value < 1.01 × 10^−6^) was used to distinguish significantly associated loci, and the different colors to distinguish different chromosomes. Specifically, the green line represents the significance threshold, while the dotted and solid gray lines indicate common signals detected by two methods and more than three methods, respectively.

To evaluate the consistency of GWAS signals across populations and to identify loci that were robust to population composition, GWAS was performed separately in the three subpopulations and in the combined population, followed by a meta-analysis of the three subpopulations. The combined-population and meta-analysis results were considered the primary sources for identifying candidate loci, whereas the population-specific GWAS results were subsequently used to evaluate cross-population consistency and population-dependent associations. All GWAS results in DP trait were used to draw the Manhattan and quantile–quantile (QQ) plots (Figure 3). The dotted gray line indicates there are two methods to detect this common signal. The solid gray line indicates there are more than three methods to detect this common signal. In total, there are 6 SNPs (there is a signal detected by CA and Meta in the same position) that were identified as significant. The relevant candidate gene names were marked in the Manhattan plot. The common position of significant signals was on chromosome 6, represented by rs37571 and rs37577 within *SEL1L3*, a protein-coding gene involved in ER-associated degradation (ERAD) and protein-folding quality control. The significant loci were mapped to several functional genes with biological relevance to muscle development and metabolism. rs5563 (Chr1) corresponds to *STRIT1*, a gene involved in potential regulation of muscle structural stability. rs127557 (Chr27) is located within *ANK1*, which plays a key role in cytoskeletal stability and function. rs95061 (Chr17) is associated with *NOC4L*, a gene involved in fundamental cellular processes such as protein synthesis and cell growth.

For LW, four significant SNPs were identified: rs12171 (Chr2), rs69377 (Chr11), rs75952 (Chr13), and rs94988 (Chr17) (Figure S5). Among these, rs94988 (Chr17) and rs12171 (Chr2) exhibited the highest proportions of variance explained (PVE = 20.96% and 91.86%, respectively), highlighting their major contributions to LW variation. rs94988 was consistently detected in the meta-analysis and combined population and is annotated to GUCY1A1, which is involved in smooth muscle relaxation and blood pressure regulation. rs12171 is annotated to GYPC, associated with red blood cell integrity and oxygen transport. Other significant loci, including PSMB7 (Chr11), involved in muscle hypertrophy, and GPR158 (Chr13), associated with endocrine regulation, showed lower PVE values.

For CW, three significant SNPs-rs106790 (Chr6), rs36257 (Chr20), and rs93045 (Chr17)-were detected, all exclusively in the combined population (Figure S6). Among these, rs106790 and rs36257 exhibited negative effects, with PVEs of 2.19% and 24.83%, respectively, and are annotated to *SNCA* (Chr6) and *DNAH5* (Chr20). *SNCA* (Chr6) encodes α-synuclein, and *DNAH5* (Chr20) encodes a dynein heavy chain; neither gene has been directly associated with carcass traits. In contrast, rs93045 (Chr17) showed a positive effect with a high PVE of 52.50% and corresponds to an unannotated long non-coding RNA, suggesting a potential regulatory role. Given its large contribution to CW variation and chromosomal location, this locus on chromosome 17 represents a key candidate region for follow-up functional studies. All significant SNPs and their positional candidate genes, annotated based on physical genomic location, are presented in Table 1, with their estimated genetic effects provided in Table S3.

Through Figure 3, the comparison of the GWAS results from individual subpopulation and whole population (whatever in CA and Meta) shows some common signals and different signals. To further assess the consistency of the detected associations across populations, we compared the directions of allelic effects for all significant SNPs in the three subpopulations (Figure S7). Most of significant SNPs showed consistent effect directions across populations, whereas only four displayed population-specific or absent effects. In particular, rs37571 and rs94988, which were jointly identified by the CA and meta-analysis approaches, showed consistent negative effects across DC, QY, and SW, providing additional support for the robustness of these associations. In contrast, SNPs such as rs9962, rs127557, rs106790, rs69377, and rs12171 showed differences in effect direction or detectable effects among populations, indicating potential population-dependent associations.

**Figure 4:**
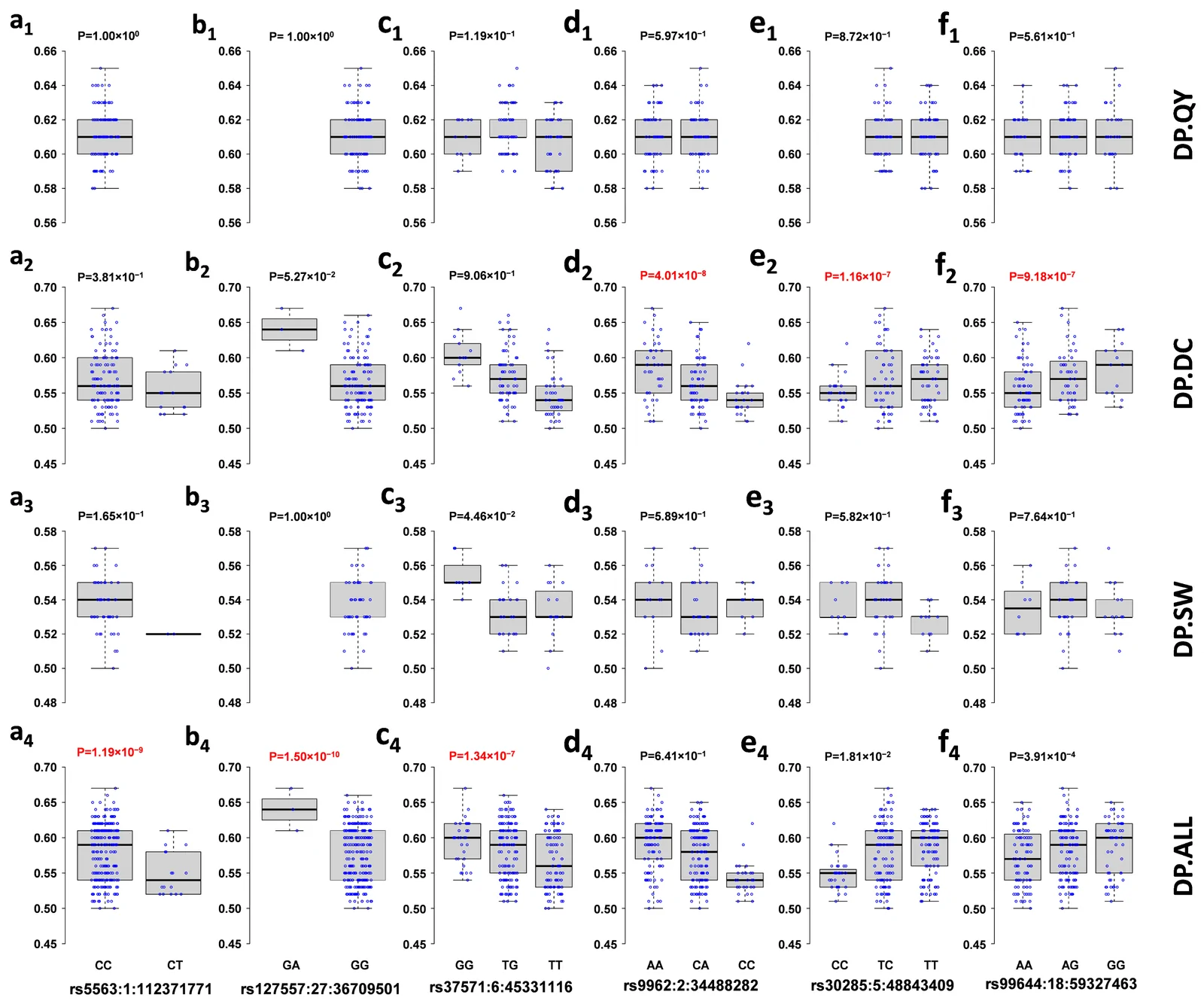
Boxplots showing the distribution of DP phenotypic values for different genotypes at each significant SNP: (**a_1_**–**a_4_**) SNP rs5563 in QY (**a_1_**), DC (**a_2_**), SW (**a_3_**), and ALL (**a_4_**); (**b_1_**–**b_4_**) SNP rs127557 in QY (**b_1_**), DC (**b_2_**), SW (**b_3_**), and ALL (**b_4_**); (**c_1_**–**c_4_**) SNP rs37571 in QY (**c_1_**), DC (**c_2_**), SW (**c_3_**), and ALL (**c_4_**); (**d_1_**–**d_4_**) SNP rs9962 in QY (**d_1_**), DC (**d_2_**), SW (**d_3_**), and ALL (**d_4_**); (**e_1_**–**e_4_**) SNP rs30285 in QY (**e_1_**), DC (**e_2_**), SW (**e_3_**), and ALL (**e_4_**); (**f_1_**–**f_4_**) SNP rs99644 in QY (**f_1_**), DC (**f_2_**), SW (**f_3_**), and ALL (**f_4_**). Each row represents a distinct population: Qingyang (QY), Dachang (DC), Sunwu (SW) and ALL (the combined dataset of all populations). Genotype classes are shown on the x-axis, and the observed DP values (%) are displayed on the y-axis. The SNP labels at the bottom of each column follow the format ‘SNP ID: Chromosome: Physical Position’. For each significant SNP, the *p*-values for that SNP in different populations are shown in red at the top of the panel. Blue circles represent phenotype values of individual samples.

To further examine the phenotypic patterns underlying these population differences, we compared the phenotypic distributions of different genotypes at the significant SNPs (Figure 4). The significant signal in such a population was marked in red. The rs5563 SNP was significant in the only whole population, while not significant in other subpopulations. That is because of there are enough samples in different genotypes in the whole population. In the subpopulation, the number of individuals with CT genotype is few or lacking. The same trend can be observed in the rs127557, and rs37571. The few or lack of the key genotype could also make fake positive (rs9962, rs30285, and rs99644). The same trend was also observed in LW and CW traits (Figures S8 and S9).

### 3.4. The LD Block Between SNPs

**Figure 5:**
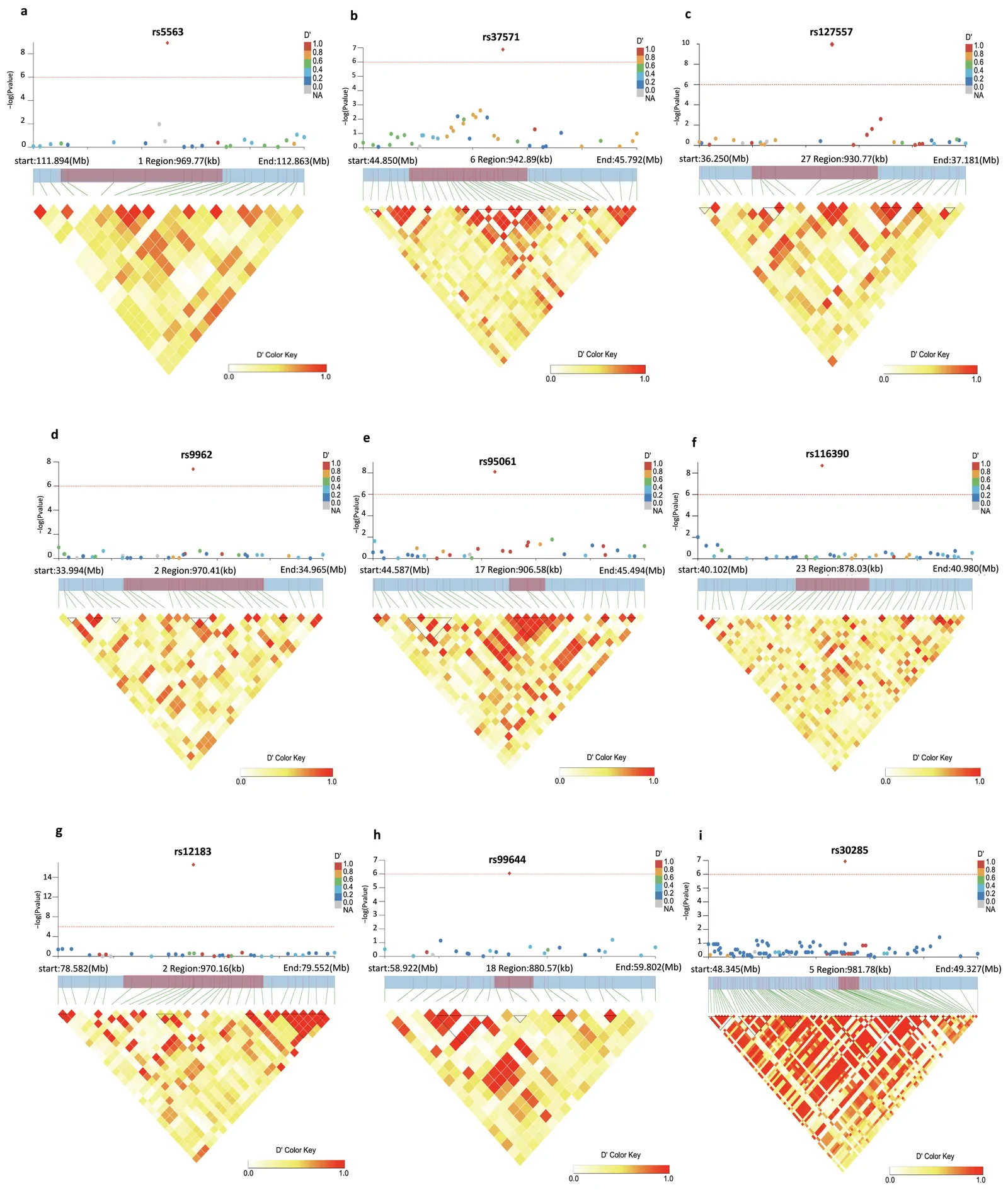
Local Manhattan plots (**top**), annotated gene regions (**middle**), and linkage disequilibrium (LD) heatmaps (**bottom**) for SNPs significantly associated with dressing percentage (DP): (**a**) rs5563; (**b**) rs37571; (**c**) rs127557; (**d**) rs9962; (**e**) rs95061; (**f**) rs116390; (**g**) rs12183; (**h**) rs99644; and (**i**) rs30285. Each panel displays a genomic region surrounding the lead SNP, with window sizes adjusted according to local SNP density and linkage disequilibrium (LD) structure. In the Manhattan plots, the red horizontal line denotes the genome-wide significance threshold (*p* < 1.01 × 10^−6^). The pink-shaded blocks represent the positions and lengths of candidate genes within the defined region, with green lines mapping individual SNPs to the pairwise LD matrix below. LD heatmaps depict pairwise D’ values, with darker red indicating stronger LD between SNPs.

To characterize the local LD patterns surrounding significant SNPs, we analyzed a 500 kb region upstream and downstream of each locus. The resulting LD heatmaps revealed distinct patterns among loci. For instance, strong LD blocks were observed around rs37571 and rs95061, indicating tight linkage with neighboring variants and highlighting their robustness as candidate loci for functional follow-up. In contrast, loci including rs5563, rs127557, rs9962, rs116390, rs12183, rs99644, and rs30285 exhibited weaker LD with adjacent markers, suggesting a more dispersed local haplotype structure. Pink-shaded regions in the heatmaps denote gene regions that either overlap with or are located near the significant SNPs (Figure 5). Similarly, loci associated with LW and CW—such as rs69377, rs12171, rs75952, rs94988, rs36257, rs93045, and rs106790—generally showed modest LD with surrounding markers (Figures S10 and S11). Overall, the observed variation in local LD patterns provides additional information for evaluating the genomic context of significant loci and prioritizing candidates for downstream functional validation.

### 3.5. Prediction with Significant Markers

In the individual population, predictive performance was used to evaluate the detection ability of the CA and meta methods. For all prediction models-including MAS and MABLUP- significant SNPs identified by each model were incorporated as covariates. Prediction accuracy was defined as the Pearson correlation between predicted gEBVs and observed phenotypes, as well as between predicted phenotypes and observed phenotypes. The former correlation assessed the predictability of gEBVs (reflecting all genetic factors), while the latter evaluated the predictability of phenotype (incorporating both genetic and non-genetic factors).

**Figure 6:**
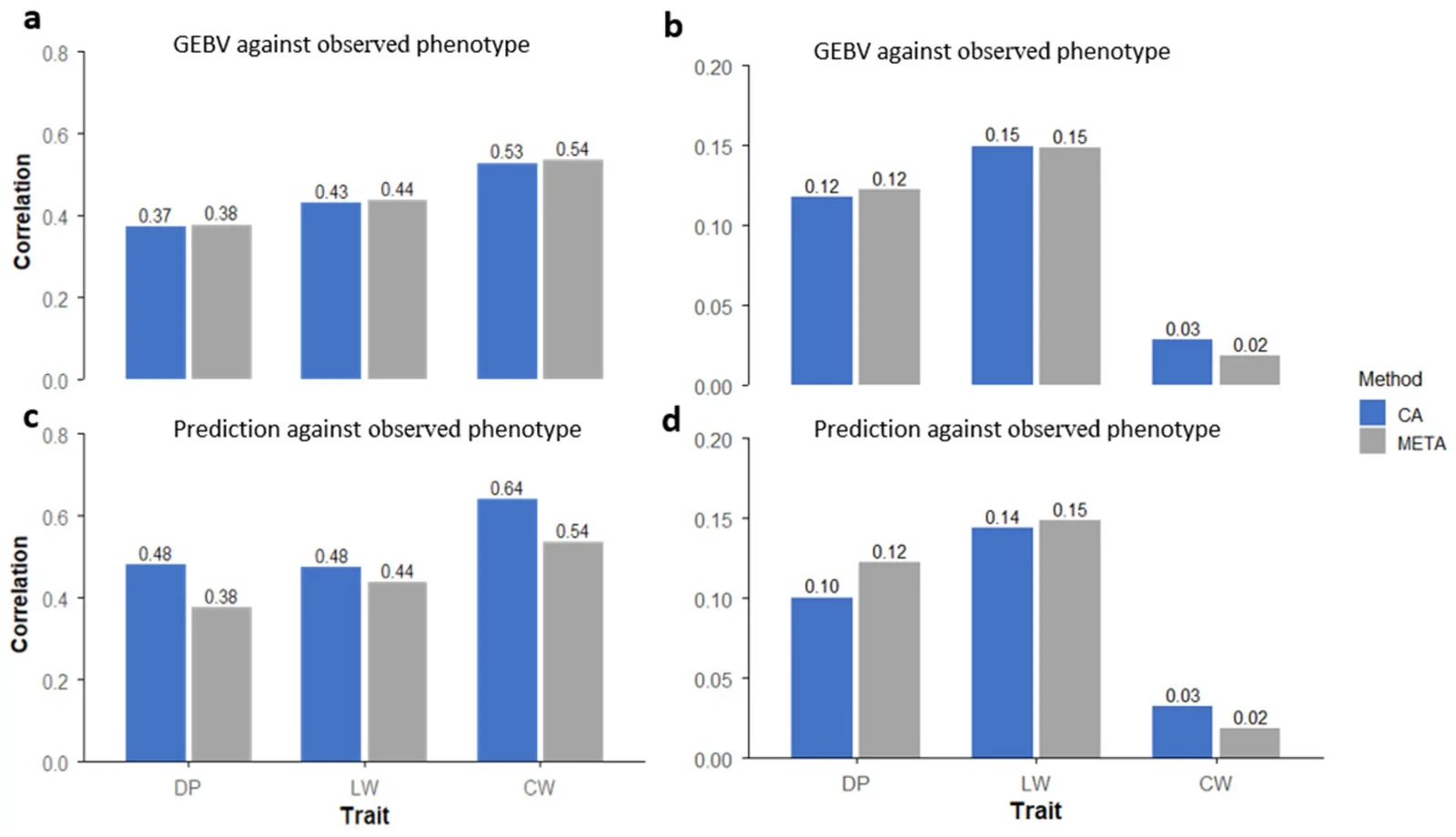
Comparison of genomic prediction performance between the CA and META models across three slaughter traits (DP, LW, CW) under two population-division strategies. Genomic selection (GS) was performed using a marker-assisted selection (MAS) framework. Panel (**a**,**c**) shows results based on the biologically structured (normal) population division, whereas panel (**b**,**d**) shows results obtained from a random population division. GEBV against observed phenotype, representing the correlation between genomic estimated breeding values (gEBVs) and observed phenotypes; Prediction against observed phenotype, representing the cross-validated correlation between predicted and observed phenotypes; Blue and grey bars represent CA and META, with correlation values labeled above each bar.

In comparisons of gEBVs accuracy, the meta approach consistently outperformed CA, with an average improvement of approximately 0.01. Notably, however, the CA model exhibited superior predictive performance in terms of phenotype accuracy. For the DP and CW traits, the improvement was 0.10, and for LW, it was approximately 0.04. This suggests that incorporating the covariance effects positively contributes to phenotype prediction accuracy (Figure 6a,c).

To further investigate whether the enhanced predictive ability stems from the population indicator used in the CA approach, the entire population was random divided into three sub-populations. The GS and GWAS procedures were then reapplied using both the CA and meta methods, generating new indicators based on random allocation. This process was repeated 20 times. As a result, the prediction accuracy of CA and meta decreased substantially, dropping to around 0.10 (Figure 6b,d).

When the kinship matrix was included in MABLUP, the meta method generally outperformed CA in prediction accuracy, with DP increasing by approximately 0.03 and LW and CW both increasing by about 0.1 (Figure S12a). When the overall population was randomly partitioned, the prediction accuracy of both meta and CA showed little difference compared with that obtained from the original population, with discrepancies within 0.01 (Figure S12b). In summary, the meta method demonstrated either superior or comparable performance overall, and the advantage associated with a predefined population indicator was diminished.

## 4. Discussion

### 4.1. Candidate Genes

In this study, we identified significant SNPs and candidate genes associated with carcass traits using CA and meta. Although only some loci were shared between the methods, their overlap provides mutual validation and highlights the polygenic and complex biological nature of carcass traits. Comparison of allelic effect directions across the three populations showed that some loci had consistent effects, whereas others showed population-specific or undetectable effects (Figure S7). Notably, rs37571 and rs94988 were jointly identified by the CA and meta-analysis approaches and showed consistent negative effects across all three populations, providing additional support for the robustness of these associations. In contrast, several loci showed different or undetectable effects among populations, which may reflect differences in allele frequencies, LD patterns, and genetic backgrounds.

For DP, *ANK1*, *STRIT1*, and *SEL1L3* represent the most biologically relevant candidates. *ANK1* encodes ankyrin 1, a membrane–cytoskeleton adaptor that contributes to the structural integrity of cell membranes. Previous studies in yak have reported associations of *ANK1*-related variants with carcass weight and meat-quality traits, including drip loss and Warner–Bratzler shear force [29,30], supporting a potential role of this gene in muscle structure and meat characteristics. *STRIT1*, associated with sarcoplasmic reticulum calcium transport, is biologically connected to muscle contraction and growth [31]. Because skeletal-muscle contraction and development are closely related to muscle mass and carcass composition, variation in *STRIT1* provides a plausible biological link to DP. *SEL1L3*, previously linked to milk protein content, was significant in both CA and meta analyses despite no known direct relation to dressing percentage [32]. Its detection in both the CA and meta-analysis results therefore provides a potentially novel candidate for carcass-related traits, although its functional relationship with DP requires further validation. Together, these three genes emerge as key candidate genes for carcass traits. Other detected genes, such as *NOC4L*, which encodes a nucleolar protein involved in ribosome biogenesis and pre-rRNA processing, potentially contributing to core cellular processes such as protein synthesis and cell growth, although functional evidence in cattle is limited [33]. *SLC4A10*, a regulator of neuronal function, was detected only in the DC population and has no known direct association with carcass traits [34]. These genes should therefore be regarded as positional or exploratory candidates rather than established functional genes.

For LW, the identified genes are primarily involved in oxygen transport, vascular regulation, and muscle protein turnover. *GYPC*, a red blood cell membrane protein, maintains cell shape and oxygen delivery, influencing muscle growth and carcass composition [35]. Adequate oxygen delivery is essential for skeletal-muscle metabolism and growth, providing a plausible physiological connection between *GYPC* variation and LW. *GUCY1A1* regulates vascular function [36], which may influence tissue perfusion and nutrient and oxygen supply. In contrast, *PSMB7* is a component of the proteasome complex and is involved in intracellular protein degradation and protein homeostasis. Its potential relationship with muscle accretion is supported by transcriptomic evidence involving the MSTN pathway [37]. Collectively, these genes suggest that variation in LW may involve not only direct regulation of muscle growth but also processes supporting muscle metabolism and protein turnover.

For CW, *SNCA* and *DNAH5* were identified as candidate genes, although their direct biological links to carcass weight are less well established. Francisco Alejandro Paredes-Sánchez et al. identified *SNCA* (α-synuclein) in Angus and Brangus cattle as associated with temperament [38], rather than with carcass traits. Similarly, *DNAH5* is critical for proper cilia function and related physiological processes [39], but both genes appear to have minimal direct impact on carcass traits. Thus, these associations may reflect population-specific signals or linkage with nearby functional variants rather than direct effects of the annotated genes. Further replication and functional studies will be necessary to determine their relevance to CW.

Moreover, several significant variants were located in long non-coding RNA regions, implying potential regulatory functions, although their biological roles in cattle remain largely unexplored. These findings collectively indicate that the identified genes were involved in diverse biological processes, including cytoskeletal organization, calcium homeostasis, vascular regulation, and protein turnover. This is consistent with recent studies showing that carcass and growth traits are influenced by multiple genomic regions and biological pathways. In 282 Ningxia Angus cattle, selection-signature analysis revealed significant enrichment of regions associated with carcass weight, marbling score, longissimus muscle area, and average daily gain, supporting the importance of multiple genomic regions in economically important traits [40]. Similarly, GWAS in Simmental cattle identified candidate genes involved in myogenesis and skeletal muscle development, highlighting the contribution of muscle-related biological processes to carcass variation [41]. Recent work in composite beef cattle populations has also demonstrated that genetic effects and genomic prediction performance can vary across heterogeneous genetic backgrounds [42]. These findings support the biological complexity of the carcass traits examined here and suggest that differences in population background may contribute to the distinct association signals identified in the present study.

### 4.2. Genomic Prediction Performance of CA and Meta

In addition to identifying candidate genes, it is crucial to assess the practical value of these genes or the genetic signals they represent in breeding. For this reason, we further compared the genomic predictive capabilities of SNPs discovered based on different GWAS strategies. The relatively similar heritability estimates across the three carcass traits (0.61–0.68; Table S2) indicated substantial additive genetic variation across traits, supporting their suitability for genomic prediction and selection.

The relatively similar heritability estimates across the three carcass traits (0.61–0.68; Table S2) indicated substantial additive genetic variation across traits, supporting their potential for genomic prediction and selection. However, similar heritability does not necessarily translate into similar prediction accuracy, as prediction performance can also be influenced by population structure, marker effects, and the extent to which the selected markers capture the underlying genetic variation. Beyond gene identification, our comparison of different genomic prediction frameworks provided important methodological insights. In MAS, when comparing gEBVs with observed phenotypes, meta-derived markers showed slightly higher correlations. However, when prediction accuracy was evaluated by correlating predicted and observed phenotypes, CA performed markedly better, suggesting that accounting for population structure may improve prediction in genetically heterogeneous populations. The markedly reduced accuracy observed in randomly divided populations is likely due to unstable SNP effects and insufficient adjustment for population structure, further highlighting the important role of correcting for population structure in genomic selection. Interestingly, meta became superior again, whereas the index provided minimal additional benefit in MABLUP. This suggests that the kinship matrix can effectively adjust for population structure even under random grouping, while the effect of the index is relatively minor, likely because already absorb most of the structure related variance, thereby reducing the incremental value of predefined groupings. Consistent with previous reports, we found that relying only on significant SNPs for prediction resulted in lower accuracy—likely because such a subset cannot capture the full genetic variation underlying complex traits [43]. Recent genomic prediction work in Australian Angus cattle further showed that prediction accuracy for growth and carcass traits was influenced by the genomic information used and the relatedness structure of the population, although increasing marker density to whole-genome sequence did not necessarily improve prediction accuracy [44]. Therefore, the contrasting performance of CA and meta under different conditions highlights their complementary strengths and implies that no single method is universally optimal. Instead, a combined or context-specific strategy may be more effective for capturing both common and population-specific genetic signals related to carcass traits.

Our results indicate that for populations with obvious geographical or genetic stratification, explicitly correcting the population structure within the model can more effectively improve the prediction accuracy of MAS. Moreover, under the MABLUP model, integrating multi-population information based on meta-analysis can capture more stable genetic effects and is robust to the choice of initial population classification.

There are several limitations that should be acknowledged. Functional validation of the identified candidate genes is still lacking, and several associated loci reside in non-coding regions where regulatory roles remain uncertain. In particular, the identified candidate SNPs have not been independently validated in other populations or through molecular approaches, which limits conclusions regarding their reproducibility and transferability. Although complementary genotype–phenotype, LD, and genomic prediction analyses provided additional evidence supporting these candidate markers, independent-population and functional validation will be necessary in future studies. Further transcriptomic, epigenomic, and gene-editing studies will be necessary to clarify the causal variants and their biological mechanisms. Additionally, the study population consisted of crossbred Angus cattle, and residual stratification or sampling bias cannot be fully ruled out despite the use of multiple correction strategies. Increasing sample size, expanding population diversity, and refining population structure modeling will be important directions for future work. Nevertheless, the integration of GWAS, meta-analysis, and genomic prediction offers a robust framework for detecting meaningful loci and evaluating their predictive value in breeding programs. The candidate genes and methodological comparisons presented here provide a valuable foundation for future molecular breeding efforts aimed at improving carcass traits and overall production efficiency in beef cattle.

## 5. Conclusions

In conclusion, this study integrated GWAS, meta-analysis, and genomic selection methodologies to uncover the genetic basis of key carcass traits in beef cattle. Some significant SNPs and biologically relevant candidate genes were identified, pointing to pathways involved in cytoskeletal organization, protein turnover and metabolic regulation. Methodologically, the comparative analyses demonstrated that meta provides improved genomic prediction under standard GS frameworks, whereas the CA approach enhances accuracy, highlighting the complementary strengths of the two strategies. Together, these findings improve our understanding of the genetic architecture of carcass traits and offer practical insights for optimizing genomic prediction models in structured populations. The candidate genes and predictive approaches identified provide a valuable foundation for future functional validation and for advancing molecular breeding programs aimed at improving carcass performance and production efficiency in beef cattle.

## Figures and Tables

**Table 1:** Significant SNPs and positional candidate genes.

SNP No. *	Chr	Position	*p* Value	Gene Name	Biotype	Trait
rs5563	1	112,371,771	1.19 × 10^−9^	*STRIT1*	protein_coding	DP
rs9962	2	34,488,282	4.01 × 10^−8^	*SLC4A10*	protein_coding	DP
rs12183	2	79,056,259	2.67 × 10^−12^	*No_name*	lncRNA	DP
rs30285	5	48,843,409	1.16 × 10^−7^	*No_name*	lncRNA	DP
rs37571	6	45,331,116	1.10 × 10^−7^	*SEL1L3*	protein_coding	DP
rs95061	17	44,994,156	7.88 × 10^−9^	*NOC4L*	protein_coding	DP
rs99644	18	59,327,463	9.18 × 10^−7^	*No_name*	Protein_coding	DP
rs116390	23	40,500,113	2.02 × 10^−9^	*No_name*	LncRNA	DP
rs127557	27	36,709,501	1.50 × 10^−10^	*ANK1*	protein_coding	DP
rs36257	6	34,851,001	3.84 × 10^−7^	*SNCA*	protein_coding	CW
rs93045	17	3,855,180	8.18 × 10^−8^	*No_name*	LncRNA	CW
rs106790	20	59,342,623	9.27 × 10^−9^	*DNAH5*	protein_coding	CW
rs12171	2	78,736,403	3.74 × 10^−7^	*GYPC*	protein_coding	LW
rs69377	11	95,429,986	2.44 × 10^−8^	*PSMB7*	protein_coding	LW
rs75952	13	26,167,866	1.54 × 10^−7^	*GPR158*	protein_coding	LW
rs94988	17	43,621,923	1.74 × 10^−10^	*GUCY1A1*	protein_coding	LW

SNP No. * indicates the sequence number in the entire tag list. Chromosomes and locations refer to physical location information in genomic data. The gene names are annotated from the GFF3 file of the Bos_taurus. ARS-UCD2.0.114 reference genome.

## Data Availability

All raw sequencing outputs were deposited in the NCBI Sequence Read Archive (SRA) under Bioproject accession PRJNA1418350. The datasets generated and/or analyzed during the current study are publicly available at https://github.com/yy0587023-rgb/Systematic-Approach-for-Compound-Angus-Populations (accessed on 2 February 2026).

## Supplementary Materials

The following supporting information can be downloaded at: https://www.mdpi.com/article/10.3390/ani16172758/s1.

## Abbreviations

The following abbreviations are used in this manuscript:

SNP	Single nucleotide polymorphism
GWAS	Genome-wide association study
GS	genomic selection
CA	Covariate Adjustment
BIC	Bayesian Information Criterion
QTNs	pseudo-quantitative trait nucleotides
BLINK	Bayesian-information and linkage-disequilibrium iteratively nested keyway
PCs	The first three principal components
CW	Carcass weight
LW	Live weight
DP	Dressing percentage
MLM	Mixed linear model
gEBVs	Genomic estimated breeding values
GBTS	Genotyping by Target Sequencing
MAF	Minor allele frequency
PCA	Principal component analysis
NJ	Neighbor-joining
GAPIT	Genome association and prediction integrated tool
MAS	Marker-Assisted Selection
MABLUP	Marker-Assisted Best Linear Unbiased Prediction
LM	Linear model

## References

[1] Jadeja R., Teng X.M., Mohan A., Duggirala K. (2022). Value-Added Utilization of Beef by-Products and Low-Value Comminuted Beef: Challenges and Opportunities. Curr. Opin. Food Sci..

[2] OECD, Food and Agriculture Organization of the United Nations (2022). OECD-FAO Agricultural Outlook 2022–2031.

[3] Lee S.-H., Park B.-H., Sharma A., Dang C.-G., Lee S.-S., Choi T.-J., Choy Y.-H., Kim H.-C., Jeon K.-J., Kim S.-D. (2014). Hanwoo Cattle: Origin, Domestication, Breeding Strategies and Genomic Selection. J. Anim. Sci. Technol..

[4] Gill J.L., Bishop S.C., McCorquodale C., Williams J.L., Wiener P. (2010). Associations between Single Nucleotide Polymorphisms in Multiple Candidate Genes and Carcass and Meat Quality Traits in a Commercial Angus-Cross Population. Meat Sci..

[5] Seabury C.M., Oldeschulte D.L., Saatchi M., Beever J.E., Decker J.E., Halley Y.A., Bhattarai E.K., Molaei M., Freetly H.C., Hansen S.L. (2017). Genome-Wide Association Study for Feed Efficiency and Growth Traits in U.S. Beef Cattle. BMC Genom..

[6] Sanchez M.-P., Tribout T., Kadri N.K., Chitneedi P.K., Maak S., Hozé C., Boussaha M., Croiseau P., Philippe R., Spengeler M. (2023). Sequence-Based GWAS Meta-Analyses for Beef Production Traits. Genet. Sel. Evol..

[7] Kolpakov V., Bukareva E., Kosyan D., Ruchay A., Ryazanov V. (2025). Identification of Candidate Genes Associated with Meat Production of Aberdeen Angus Cattle. Animals.

[8] Baneh H., Elatkin N., Gentzbittel L. (2025). Genome-Wide Association Studies and Genetic Architecture of Carcass Traits in Angus Beef Cattle Using Imputed Whole-Genome Sequences Data. Genet. Sel. Evol..

[9] Jiang J., Ma L., Prakapenka D., VanRaden P.M., Cole J.B., Da Y. (2019). A Large-Scale Genome-Wide Association Study in U.S. Holstein Cattle. Front. Genet..

[10] Zhu H., Li X., Zhang M., Liu S., Zhang Y., Zheng Y., Wei Z., Han M., Huang H., Fu T. (2025). Detection of Selection Signatures and Genome-Wide Association Analysis of Body Weight Traits in Xianan Cattle. Genes.

[11] Adhikari M., Kantar M.B., Longman R.J., Lee C.N., Oshiro M., Caires K., He Y. (2023). Genome-Wide Association Study for Carcass Weight in Pasture-Finished Beef Cattle in Hawai’i. Front. Genet..

[12] Liu Y., Qi X., La Y., Ma X., Ren W., Yang G., Zhang Z., Chu M., Wu X., Guo X. (2026). Genome-Wide Association Study of Gross Hair Weight and Hair Length Traits in Different Body Regions of Tianzhu White Yak. Biomolecules.

[13] Zeggini E., Ioannidis J.P. (2009). Meta-Analysis in Genome-Wide Association Studies. Pharmacogenomics.

[14] Huang M., Liu X., Zhou Y., Summers R.M., Zhang Z. (2019). BLINK: A Package for the next Level of Genome-Wide Association Studies with Both Individuals and Markers in the Millions. GigaScience.

[15] Neupane B. (2025). Systematic Comparison of GWAS Methods in Wheat: Balancing Statistical Power, False Positive Control, and Computational Efficiency. Preprints.

[16] Shadish W.R. (2015). Introduction to the Special Issue on the Origins of Modern Meta-analysis. Res. Synth. Methods.

[17] Willer C.J., Li Y., Abecasis G.R. (2010). METAL: Fast and Efficient Meta-Analysis of Genomewide Association Scans. Bioinformatics.

[18] Wakchaure R., Ganguly S. (2015). Marker Assisted Selection (MAS) in Animal Breeding: A Review. J. Drug Metab. Toxicol..

[19] Hay E.H. (2024). Machine Learning for the Genomic Prediction of Growth Traits in a Composite Beef Cattle Population. Animals.

[20] Zhang Z., Shi S., Zhang Q., Aamand G.P., Lund M.S., Su G., Ding X. (2023). Improving Genomic Prediction Accuracy in the Chinese Holstein Population by Combining with the Nordic Holstein Reference Population. Animals.

[21] Browning S.R., Browning B.L. (2007). Rapid and Accurate Haplotype Phasing and Missing-Data Inference for Whole-Genome Association Studies By Use of Localized Haplotype Clustering. Am. J. Hum. Genet..

[22] Wang J., Zhang Z. (2026). GAPIT Version 4: Integration of GWAS into Genomic Prediction. Mol. Biol. Evol..

[23] Zhang C., Dong S.-S., Xu J.-Y., He W.-M., Yang T.-L. (2019). PopLDdecay: A Fast and Effective Tool for Linkage Disequilibrium Decay Analysis Based on Variant Call Format Files. Bioinformatics.

[24] Bradbury P.J., Zhang Z., Kroon D.E., Casstevens T.M., Ramdoss Y., Buckler E.S. (2007). TASSEL: Software for Association Mapping of Complex Traits in Diverse Samples. Bioinformatics.

[25] Dong S.-S., He W.-M., Chen J.-J., Zhang C., Blauwendraat C., Ding J.-H., Yang T.-L. (2021). LDBlockShow: A Fast and Convenient Tool for Visualizing Linkage Disequilibrium and Haplotype Blocks Based on Variant Call Format Files. Brief. Bioinform..

[26] Guo Z., Yang Q., Huang F., Zheng H., Sang Z., Xu Y., Zhang C., Wu K., Tao J., Prasanna B.M. (2021). Development of High-Resolution Multiple-SNP Arrays for Genetic Analyses and Molecular Breeding through Genotyping by Target Sequencing and Liquid Chip. Plant Commun..

[27] Zhang W., Gao X., Zhang Y., Zhao Y., Zhang J., Jia Y., Zhu B., Xu L., Zhang L., Gao H. (2018). Genome-Wide Assessment of Genetic Diversity and Population Structure Insights into Admixture and Introgression in Chinese Indigenous Cattle. BMC Genet..

[28] Mulim H.A., Campos G.S., Cardoso F.F., Pedrosa V.B., Latimer K., Upperman L.R., Knowles A.J., Garcia A., Retallick K., Miller S. (2026). Genomic Structure and Selection History across Angus Populations Worldwide: Insights from ROH, Selection Mapping, and Functional Analyses. Mamm. Genome.

[29] Giacomello E., Quarta M., Paolini C., Squecco R., Fusco P., Toniolo L., Blaauw B., Formoso L., Rossi D., Birkenmeier C. (2015). Deletion of Small Ankyrin 1 (sAnk1) Isoforms Results in Structural and Functional Alterations in Aging Skeletal Muscle Fibers. Am. J. Physiol.-Cell Physiol..

[30] Hu J., Gao X., Shi B., Chen H., Zhao Z., Wang J., Liu X., Li S., Luo Y. (2021). Sequence and Haplotypes of Ankyrin 1 Gene (ANK1) and Their Association with Carcass and Meat Quality Traits in Yak. Mamm. Genome.

[31] Veluri R., Pollin G., Wagenknecht J.B., Urrutia R., Zimmermann M.T. (2025). An Integrative Multitiered Computational Analysis for Better Understanding the Structure and Function of 85 Miniproteins. NAR Genom. Bioinform..

[32] Sanchez M.-P., Ramayo-Caldas Y., Wolf V., Laithier C., El Jabri M., Michenet A., Boussaha M., Taussat S., Fritz S., Delacroix-Buchet A. (2019). Sequence-Based GWAS, Network and Pathway Analyses Reveal Genes Co-Associated with Milk Cheese-Making Properties and Milk Composition in Montbéliarde Cows. Genet. Sel. Evol..

[33] Zhu X., Zhang W., Guo J., Zhang X., Li L., Wang T., Yan J., Zhang F., Hou B., Gao N. (2019). Noc4L-Mediated Ribosome Biogenesis Controls Activation of Regulatory and Conventional T Cells. Cell Rep..

[34] Ding W., Gong W., Bou T., Shi L., Lin Y., Shi X., Li Z., Wu H., Dugarjaviin M., Bai D. (2025). Analysis of ROH Characteristics Across Generations in Grassland-Thoroughbred Horses and Identification of Loci Associated with Athletic Traits. Animals.

[35] Jiang A., Zhang J., Yuan C.-B., Xiang B.-J., Huang D.-J., Gao L.-F., Guang-Xin E. (2019). Whole-Genome Scanning for the Heat-Resistance-Associated Genes in the Droughtmaster Breed (*Bos taurus*). 3 Biotech.

[36] Childers K.C., Garcin E.D. (2018). Structure/Function of the Soluble Guanylyl Cyclase Catalytic Domain. Nitric Oxide.

[37] Sadkowski T., Jank M., Zwierzchowski L., Siadkowska E., Oprządek J., Motyl T. (2008). Gene Expression Profiling in Skeletal Muscle of Holstein-Friesian Bulls with Single-Nucleotide Polymorphism in the Myostatin Gene 5′-Flanking Region. J. Appl. Genet..

[38] Paredes-Sánchez F.A., Sifuentes-Rincón A.M., Lara-Ramírez E.E., Casas E., Rodríguez-Almeida F.A., Herrera-Mayorga E.V., Randel R.D. (2022). Identificación de Genes Candidatos y SNP Relacionados Con El Temperamento Del Ganado Utilizando Un Análisis GWAS Junto Con Un Análisis de Redes Interactuantes. Rev. Mex. Cienc. Pecu..

[39] Dementieva N.V., Dysin A.P., Shcherbakov Y.S., Nikitkina E.V., Musidray A.A., Petrova A.V., Mitrofanova O.V., Plemyashov K.V., Azovtseva A.I., Griffin D.K. (2024). Risk of Sperm Disorders and Impaired Fertility in Frozen–Thawed Bull Semen: A Genome-Wide Association Study. Animals.

[40] Yin H., Feng Y., Wang Y., Jiang Q., Zhang J., Zhao J., Chen Y., Wang Y., Peng R., Wang Y. (2024). Genome-Wide Scans for Selection Signatures in Ningxia Angus Cattle Reveal Genetic Variants Associated with Economic and Adaptive Traits. Animals.

[41] Bordbar F., Mohammadabadi M., Jensen J., Xu L., Li J., Zhang L. (2022). Identification of Candidate Genes Regulating Carcass Depth and Hind Leg Circumference in Simmental Beef Cattle Using Illumina Bovine Beadchip and Next-Generation Sequencing Analyses. Animals.

[42] Deng T., Li K., Du L., Liang M., Qian L., Xue Q., Qiu S., Xu L., Zhang L., Gao X. (2024). Genome-Wide Gene–Environment Interaction Analysis Identifies Novel Candidate Variants for Growth Traits in Beef Cattle. Animals.

[43] Kamprasert N., Aliloo H., Van Der Werf J.H.J., Duff C.J., Clark S.A. (2025). Effect of Using Preselected Markers from Imputed Whole-Genome Sequence for Genomic Prediction in Angus Cattle. Genet. Sel. Evol..

[44] Kamprasert N., Aliloo H., Van Der Werf J.H.J., Duff C.J., Clark S.A. (2025). Genomic Prediction Using Imputed Whole-Genome Sequence Data in Australian Angus Cattle. J. Anim. Breed. Genet..

