# Supplementary material for: Systematic Approach for Compound Angus Populations Revealing Positional Candidate Genes and Improving Prediction Accuracy in Carcass Traits
Source: Animals (Basel). 2026 Sep 2;16(17):2758. doi: 10.3390/ani16172758 (PMC13565728; doi:10.3390/ani16172758)
Supplement: Supplementary file 1 [file animals-16-02758-s001.zip › animals-4465922-supplementary.pdf]

Supplementary Material

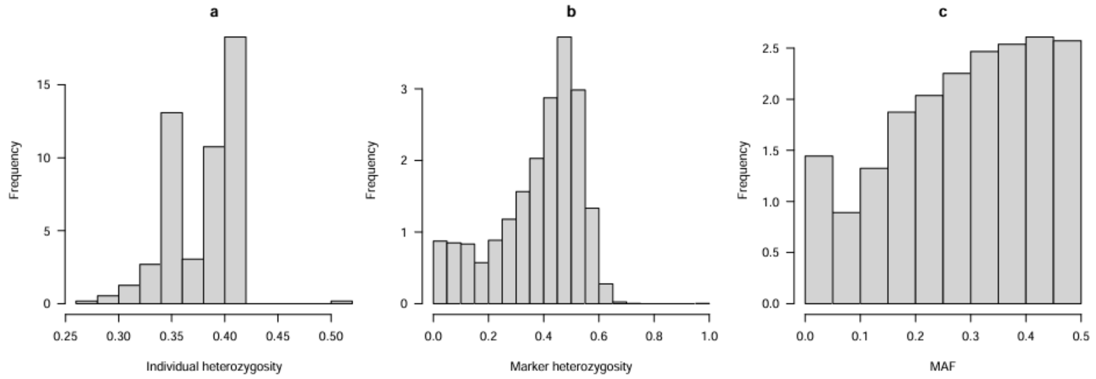

Figure S1 Heterozygosity and Minor Allele Frequency. This figure shows the frequency distribution of heterozygosity and minor allele frequency (MAF) for cattle individuals and genomic markers.

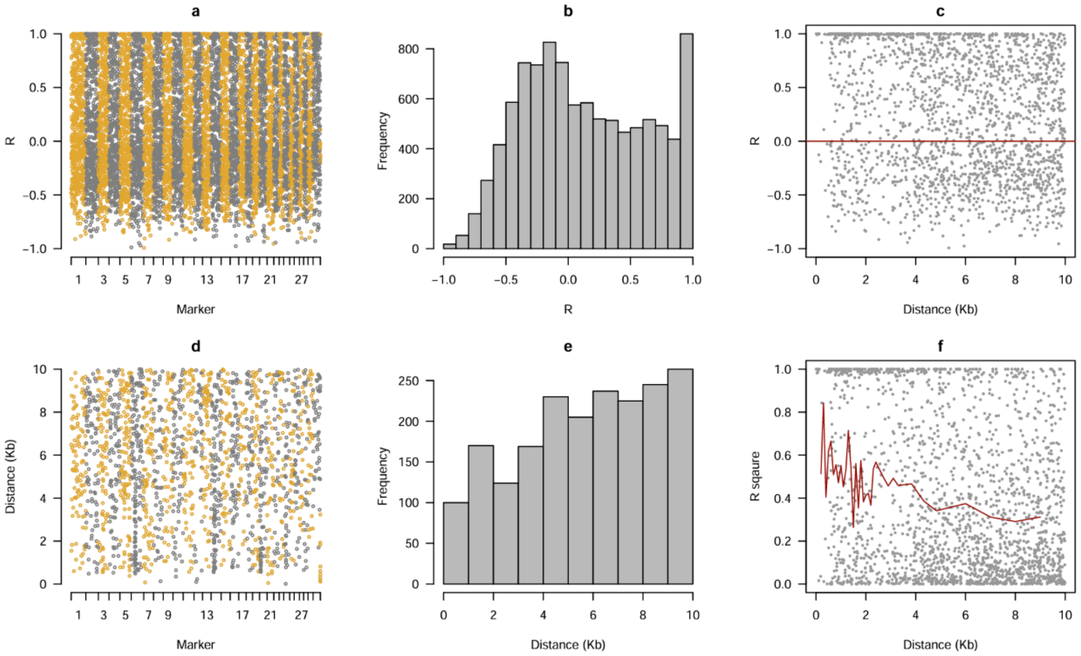

Figure S2 Visualization of genetic marker relationships and linkage disequilibrium (LD) patterns.(A) Heatmap of pairwise Pearson correlation coefficients (R) among genetic markers, with marker positions on the x- and y-axes;(B) Frequency distribution of pairwise correlation coefficients (R) across all marker pairs;(C) Scatter plot of pairwise correlation coefficients (R) against physical distance (Kb) between markers; the red horizontal line indicates no correlation (R = 0);(D) Heatmap of physical distances (Kb) between genetic markers, with marker positions on both axes;(E) Frequency distribution of pairwise physical distance intervals (Kb) among markers;(F) Scatter plot of squared correlation coefficients (R^2) versus physical distance (Kb) between markers; the red trend line depicts LD decay with increasing genomic distance.

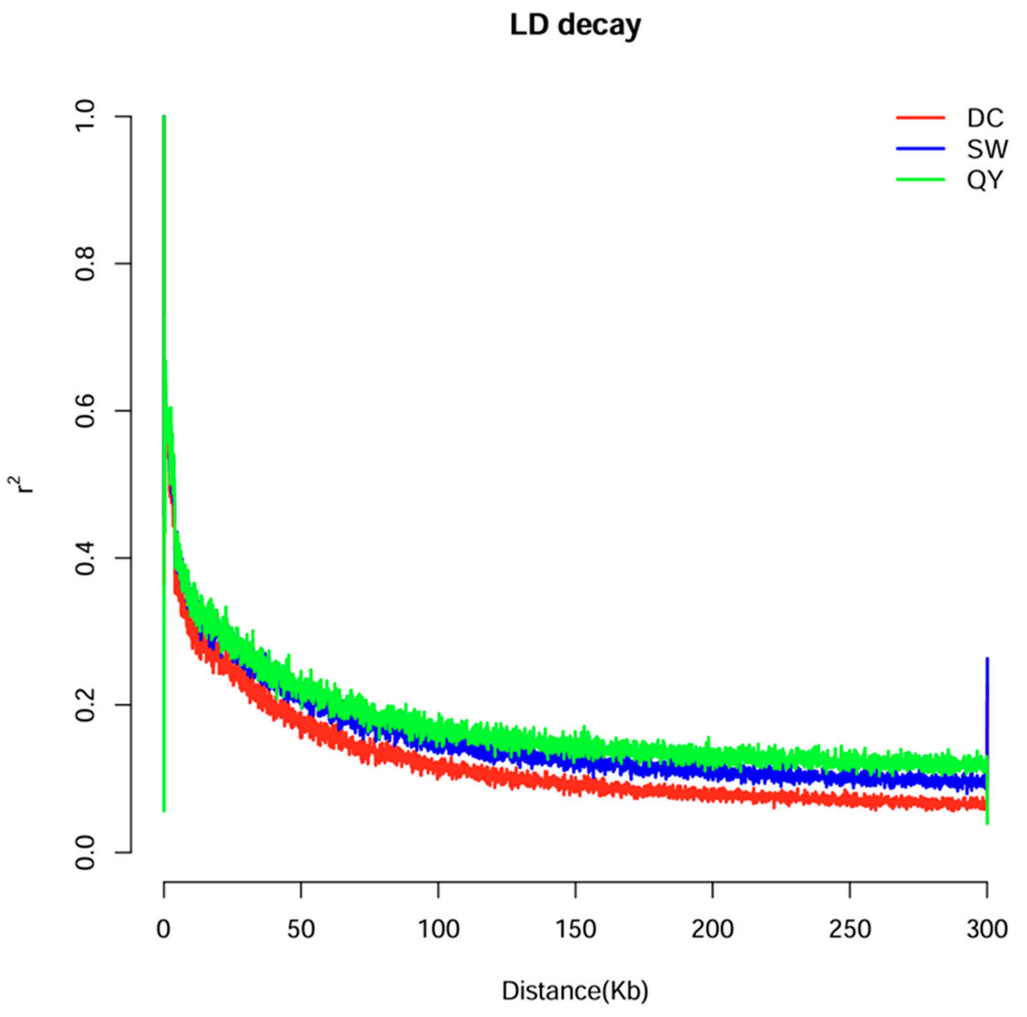

Figure S3 LD decay curves across three populations (Qingyang (QY), Dachang (DC)and Sunwu (SW)). The LD between SNP markers, measured by r^2 ,is plotted against their physical distance (Kb). The LD decay curves were generated using PopLDdecay-3.42, which calculates genome-wide LD and summarizes its decay with increasing inter-marker distance.

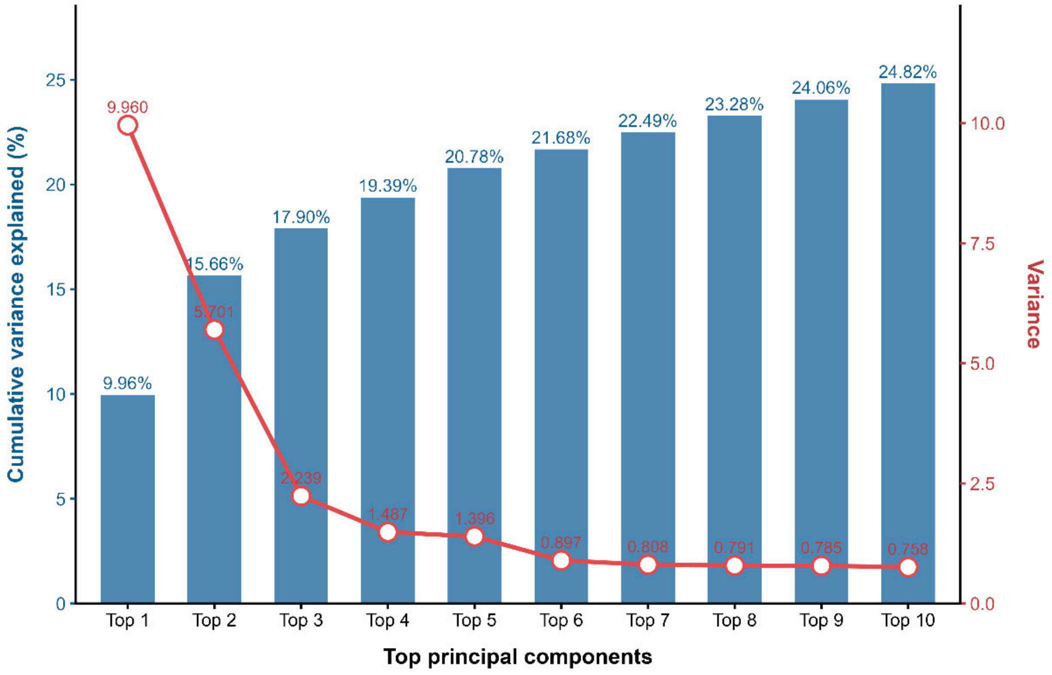

Figure S4. Principal component analysis (PCA) of genome-wide SNP genotypes. Blue bars represent the cumulative percentage of total genetic variance explained by the first 10 principal components, while the red line represents the corresponding variance of each individual principal component.

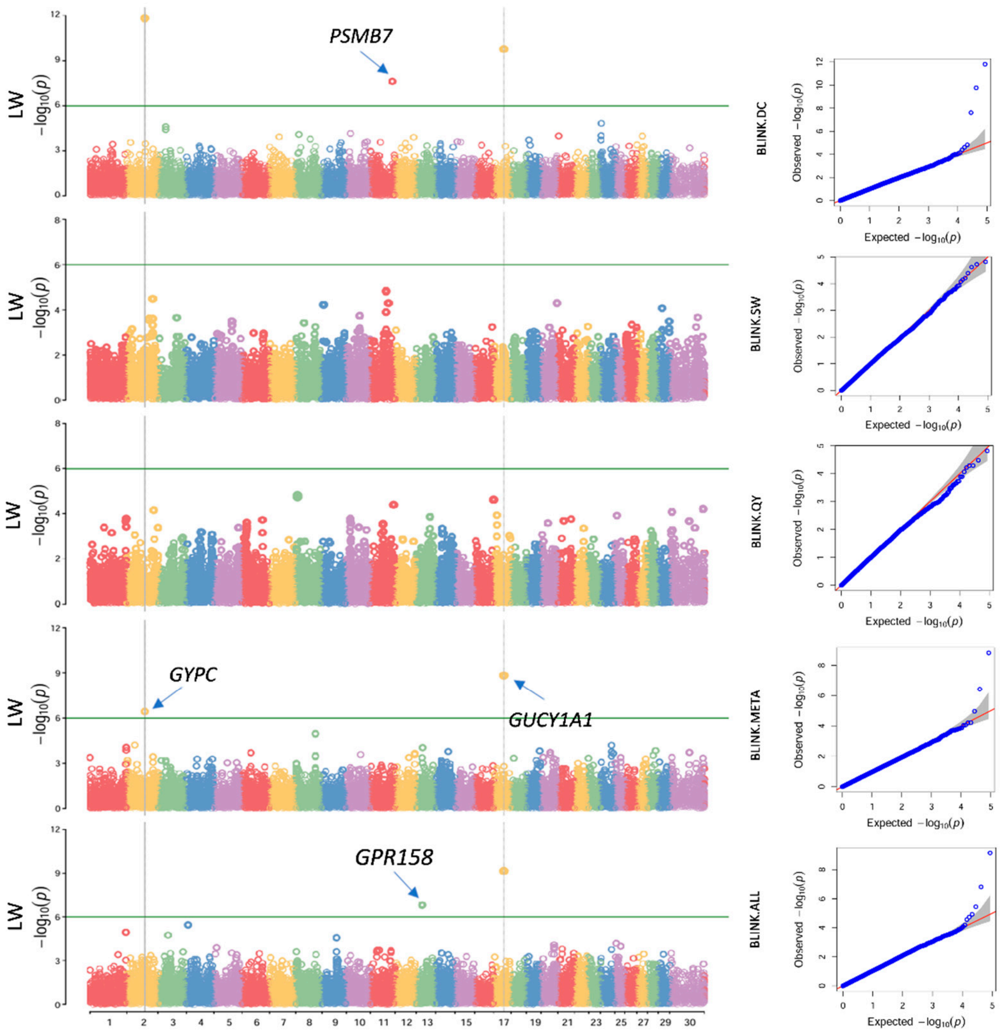

Figure S5 Manhattan and quantile–quantile (QQ) plots of the p-values for the genome-wide association study of the LW trait in cattle, performed separately for the Sunwu, Dachang, and Qingyang populations, for the combined population, and for the meta-analysis, based on the BLINK method； the horizontal line of significance threshold (p-values $< 1.01 \times 10^{-6}$) was used to distinguish significantly associated loci, and the different colors to distinguish different chromosomes. Specifically, the green line represents the significance threshold, while the dotted and solid gray lines indicate common signals detected by two methods and more than three methods, respective-ly.

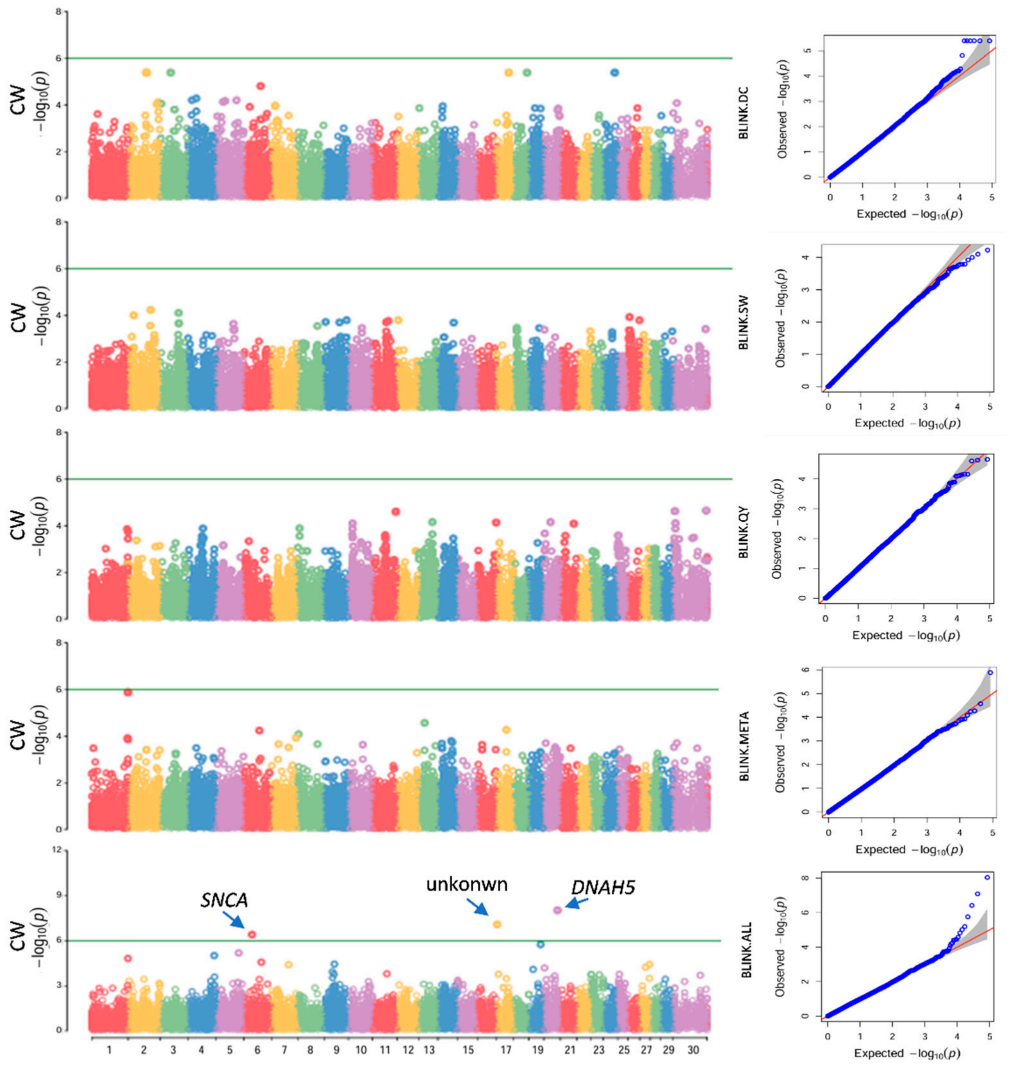

Figure S6 Manhattan and quantile–quantile (QQ) plots of the p-values for the genome-wide association study of the CW trait in cattle, performed separately for the Sunwu, Dachang, and Qingyang populations, for the combined population, and for the meta-analysis, based on the BLINK method； the horizontal line of significance threshold (p-values < 1.01 × 10^{-6}) was used to distinguish significantly associated loci, and the different colors to distinguish different chromosomes. Specifically, the green line represents the significance threshold, while the dotted and solid gray lines indicate common signals detected by two methods and more than three methods, respective-ly.

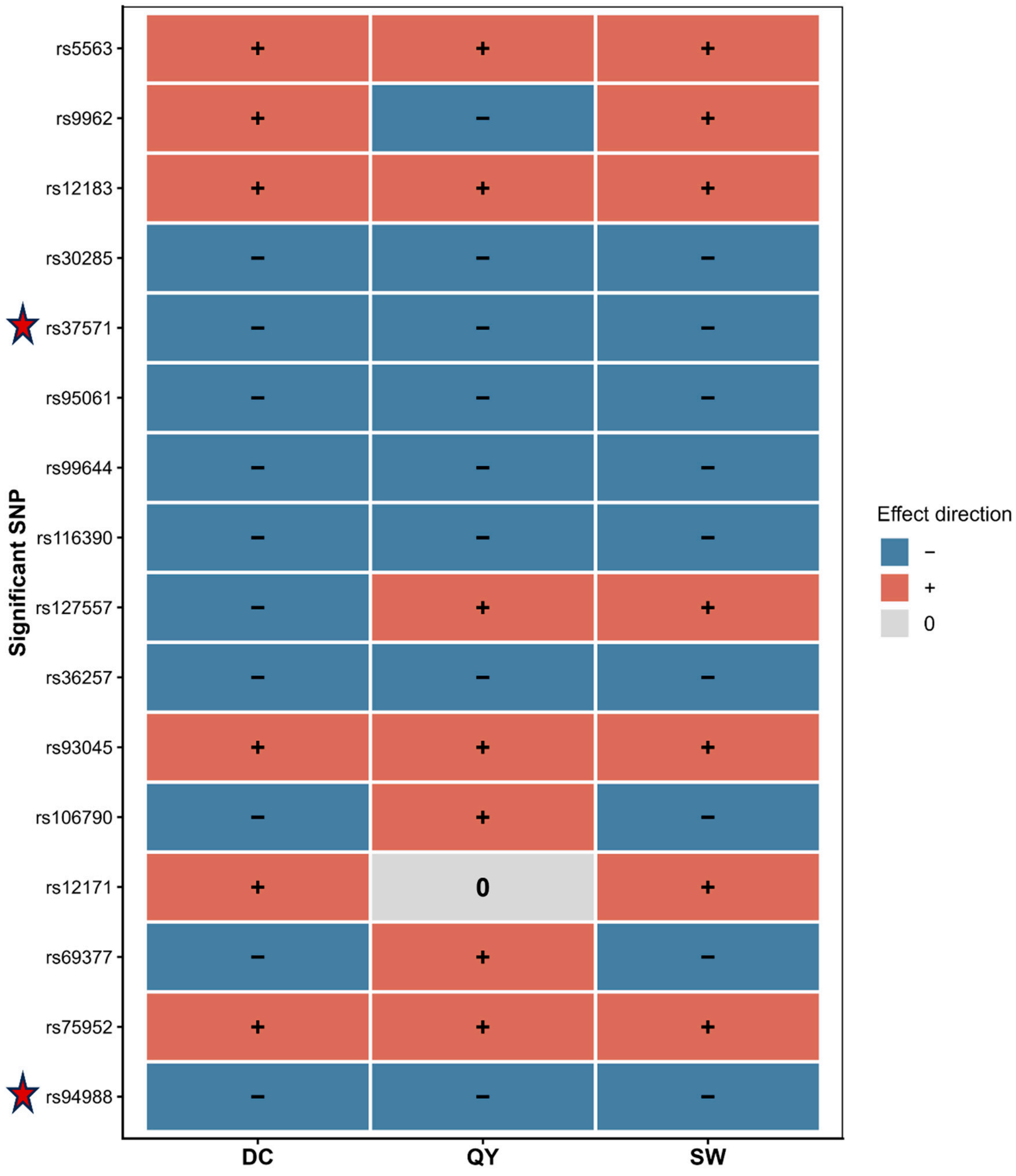

Figure S7. Consistency of allelic effect directions for significant SNPs across the three cattle populations (DC, SW, and QY). Each cell represents the estimated allelic effect direction of a significant SNP in the corresponding population, with "+" and "–" indicating positive and negative effects, respectively, and "0" indicating no detectable effect. Red five-point stars indicate SNPs jointly identified by the CA and meta-analysis approaches. Consistent effect directions across populations support the robustness of the detected associations.

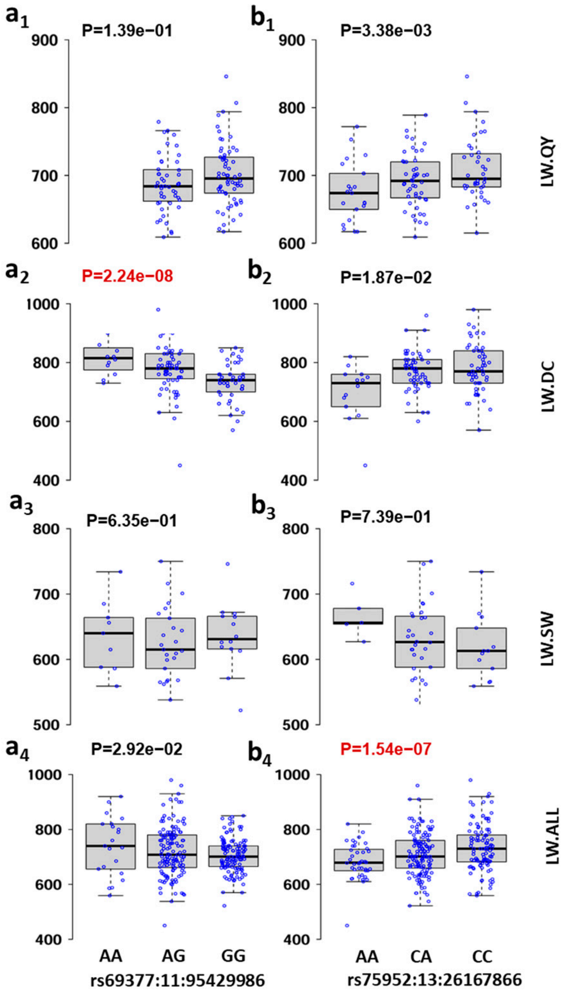

Figure S8 Boxplots showing the distribution of LW phenotypic values for different genotypes at each significant SNP: (a_1–a_4) SNP rs69377 in QY (a_1), DC (a_2), SW (a_3), and ALL (a_4); (b_1–b_4) SNP rs75952 in QY (b_1), DC (b_2), SW (b_3), and ALL (b_4). Each row represents a distinct population: Qingyang (QY), Dachang (DC), Sunwu (SW) and ALL (the combined dataset of all populations). Genotype classes are shown on the x-axis, and the observed LW values (kg) are displayed on the y-axis. The SNP labels at the bottom of each column follow the format 'SNP ID: Chromosome: Physical Position'. For each significant SNP, the p-values for that SNP in different populations are shown in red at the top of the panel. Blue circles represent phenotype values of individual samples.

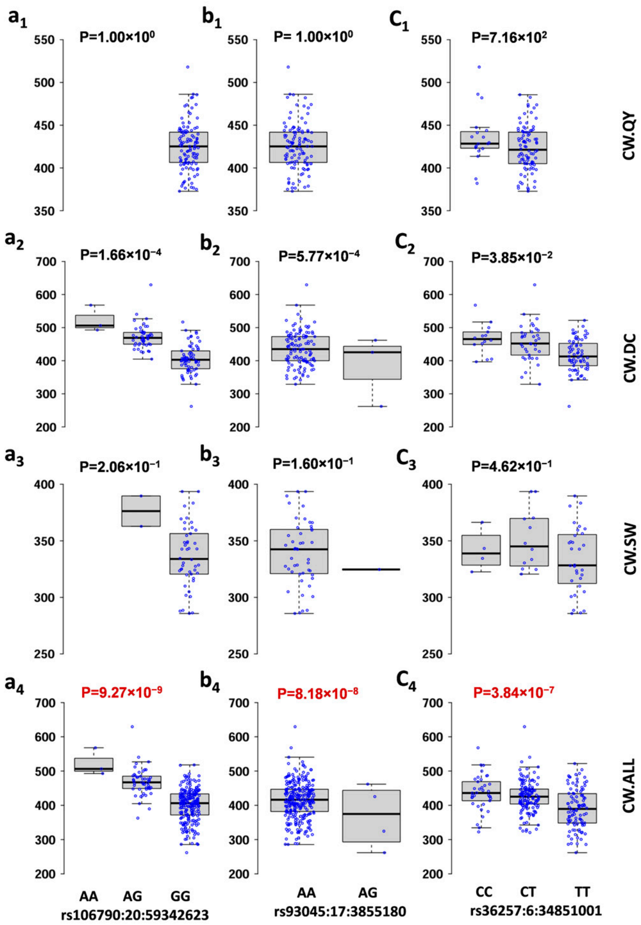

Figure S9 Boxplots showing the distribution of CW phenotypic values for different genotypes at each significant SNP: (a_1–a_4) SNP rs106790 in QY (a_1), DC (a_2), SW (a_3), and ALL (a_4); (b_1–b_4) SNP rs93045 in QY (b_1), DC (b_2), SW (b_3), and ALL (b_4); (c_1–c_4) SNP rs36257 in QY (c_1), DC (c_2), SW (c_3), and ALL (c_4). Each row represents a distinct population: Qingyang (QY), Dachang (DC), Sunwu (SW) and ALL (the combined dataset of all populations). Genotype classes are shown on the x-axis, and the observed CW values (kg) are displayed on the y-axis. The SNP labels at the bottom of each column follow the format 'SNP ID: Chromosome: Physical Position'. For each significant SNP, the p-values for that SNP in different populations are shown in red at the top of the panel. Blue circles represent phenotype values of individual samples.

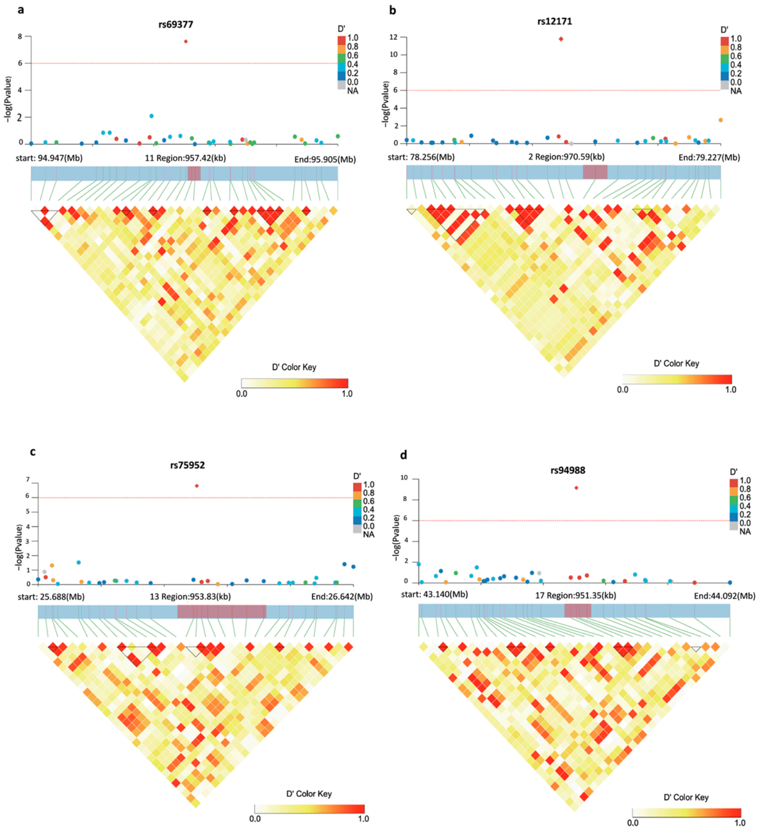

Figure S10 Local Manhattan plots (top), annotated gene regions (middle), and linkage disequilibrium (LD) heatmaps (bottom) for SNPs significantly associated with LW: (a) rs69377; (b) rs12171; (c) rs75952; (d) rs94988. Each panel shows the genomic region surrounding the lead SNP, with window sizes adjusted according to local SNP density and LD patterns. In the Manhattan plots, the red horizontal line represents the genome-wide significance threshold ($p < 1.01 \times 10^{-6}$). The middle panel displays the annotated gene structures within the targeted genomic interval; the pink shaded blocks indicate the gene regions overlapping or adjacent to the lead SNP, highlighting potential candidate genes. The LD heatmaps illustrate pairwise D′ values among SNPs in the region, where darker red indicates stronger LD.

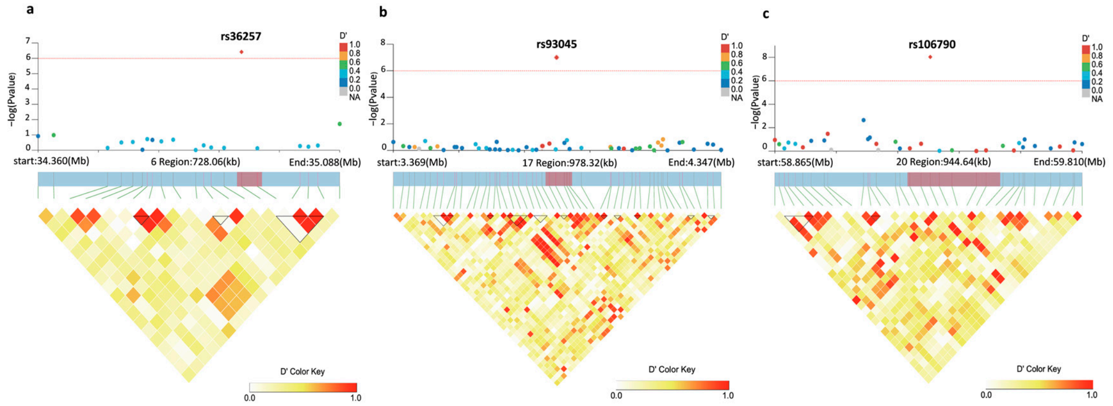

Figure S11 Local Manhattan plots (top), annotated gene regions (middle), and linkage disequilibrium (LD) heatmaps (bottom) for SNPs significantly associated with CW: (a) rs36257; (b) rs93045; (c) rs106790. Each panel shows the genomic region surrounding the lead SNP, with window sizes adjusted according to local SNP density and LD patterns. In the Manhattan plots, the red horizontal line represents the genome-wide significance threshold ($p < 1.01 \times 10^{-6}$). The middle panel displays the annotated gene structures within the targeted genomic interval; the pink shaded blocks indicate the gene regions overlapping or adjacent to the lead SNP, highlighting potential candidate genes. The LD heatmaps illustrate pairwise D′ values among SNPs in the region, where darker red indicates stronger LD.

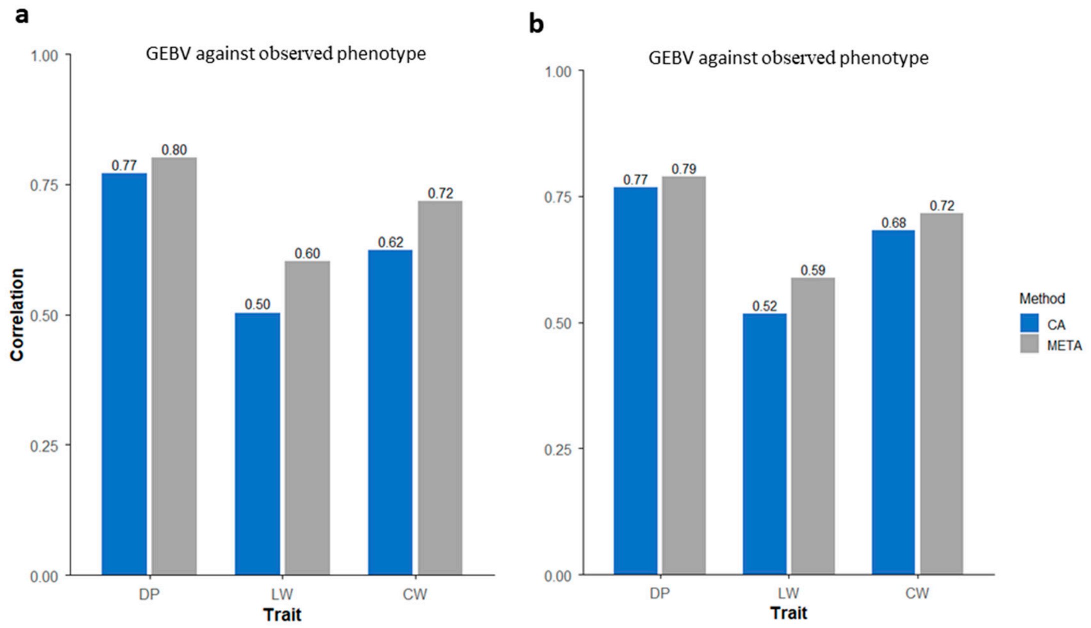

Figure S12 Comparison of genomic prediction performance between the CA and META models across three slaughter traits (DP, LW, CW) under two population-division strategies. Genomic selection (GS) was performed using an MABLUP framework. Panel (a) shows results based on the biologically structured (normal) population division, whereas panel (b) shows results obtained from a random population division. GEBV against observed phenotype, representing the correlation between genomic estimated breeding values (gEBVs) and observed phenotypes. Blue and grey bars represent CA and META, with correlation values labeled above each bar.

Table S1 Descriptive statistics of carcass traits across three distinct cattle populations.

Trait	Population	Sample Size (N)	Mean ± SE	Range (Min – Max)
LW	DC	122	766.39 ± 7.22	450.00 – 980.00
	QY	109	695.41 ± 4.24	609.00 – 846.00
	SW	48	630.60 ± 7.60	522.00 – 750.00
CW	DC	122	434.70 ± 4.85	262.00 – 630.00
	QY	109	425.33 ± 2.65	373.00 – 518.00
	SW	48	338.39 ± 4.10	286.00 – 394.00
DP	DC	122	56.74 ± 0.34	58.00 – 65.00
	QY	109	61.18 ± 0.14	50.00 – 67.00
	SW	48	53.62 ± 0.22	50.00 – 57.00

LW, live weight (kg); CW, carcass weight (kg); DP, dressing percentage (%); DC, Dachang population; QY, Qingyang population; SW, Sunwu population; SE, Standard Error.

Table S2 Variance component estimates and narrow-sense heritability (± SE) for three carcass traits

Trait	VG ± SE	VE ± SE	VP	h^2 ± SE	95% CI of h^2
DP	3.95 ± 0.47	1.98 ± 0.24	5.922	0.67 ± 0.04	[0.59, 0.74]
CW	874.91 ± 105.14	565.35 ± 68.43	1,440.26	0.61 ± 0.04	[0.53, 0.69]
LW	2909.35 ± 349.61	1345.97 ± 162.92	4,255.31	0.68 ± 0.04	[0.61, 0.76]

DP, dressing percentage; CW, carcass weight; LW, loin weight; VG, additive genetic variance; VE, residual variance; VP, phenotypic variance (VG + VE); h^2, narrow-sense heritability (VG/VP); SE, standard error; CI, Wald-type confidence interval truncated to [0, 1].

Variance components were estimated via restricted maximum likelihood (REML) using the GAPIT mixed linear model (MLM). SEs were calculated using Kruijer's conservative asymptotic REML approximation combined with the delta method for h^2.

Table S3 Significant SNPs identified by the CA and meta-analysis approaches method and their estimated genetic effects

SNP No. *	Chr	position	PVE（%）	Effect	method
rs5563	1	112,371,771	16.10493	0.031397	CA
rs9962	2	34,488,282	2.68955	0.010565	CA
rs12183	2	79,056,259	44.35025	0.040103	Meta
rs30285	5	48,843,409	6.235688	-0.01081	CA
rs37571	6	45,331,116	2.213864	-0.00847	Meta/CA
rs95061	17	44,994,156	4.25656	-0.01737	Meta
rs99644	18	59,327,463	6.18016	-0.00944	CA
rs116390	23	40,500,113	20.51488	-0.01499	Meta
rs127557	27	36,709,501	68.05572	-0.06509	CA
rs36257	6	34,851,001	2.190922	-12.7971	CA
rs93045	17	3,855,180	52.50343	72.74001	CA
rs106790	20	59,342,623	24.82766	-35.9071	CA
rs12171	2	78,736,403	91.86077	388.8639	Meta
rs69377	11	95,429,986	1.55319	-43.2099	CA
rs75952	13	26,167,866	7.003888	19.71486	CA
rs94988	17	43,621,923	20.96367	-30.172	Meta/CA

SNP No.* indicates the sequence number in the entire tag list. Chromosomes and locations refer to physical location information in genomic data. PVE (Phenotypic Variance Explained) denotes the proportion of phenotypic variance explained by each SNP. Effect refers to represents the estimated allelic effect. Method indicates the analysis approach used to identify the significant SNP, with CA denoting the covariate-adjusted analysis and Meta denoting the meta-analysis. Meta/CA indicates that the SNP was identified by both approaches.
